# Immune responses in Omicron SARS-CoV-2 breakthrough infection in vaccinated adults

**DOI:** 10.1038/s41467-022-31888-y

**Published:** 2022-07-18

**Authors:** Hassen Kared, Asia-Sophia Wolf, Amin Alirezaylavasani, Anthony Ravussin, Guri Solum, Trung The Tran, Fridtjof Lund-Johansen, John Torgils Vaage, Lise Sofie Nissen-Meyer, Unni C. Nygaard, Olav Hungnes, Anna H. Robertson, Lisbeth Meyer Næss, Lill Trogstad, Per Magnus, Ludvig A. Munthe, Siri Mjaaland

**Affiliations:** 1 KG Jebsen Centre for B cell malignancy, Institute of Clinical medicine, University of Oslo, Oslo, Norway; 2 Department of Immunology, Oslo University Hospital, Oslo, Norway; 3 Division of Infection Control, Norwegian Institute of Public Health, Oslo, Norway; 4 ImmunoLingo Convergence Center, Institute of Clinical medicine, University of Oslo, Oslo, Norway; 5 Center for Fertility and Health, Norwegian Institute of Public Health, Oslo, Norway

**Keywords:** SARS-CoV-2, Viral host response, Viral infection

## Abstract

The SARS-CoV-2 Omicron variant has more than 15 mutations in the receptor binding domain of the Spike protein enabling increased transmissibility and viral escape from antibodies in vaccinated individuals. It is unclear how vaccine immunity protects against Omicron infection. Here we show that vaccinated participants at a super-spreader event have robust recall response of humoral and pre-existing cellular immunity induced by the vaccines, and an emergent de novo T cell response to non-Spike antigens. Individuals with Omicron SARS-CoV-2 breakthrough infections have significantly increased activated SARS-CoV-2 wild type Spike-specific cytotoxic T cells, activated follicular helper (T_FH_) cells, functional T cell responses, boosted humoral responses, and rapid release of Spike and RBD-specific IgG^+^ B cell plasmablasts and memory B cells into circulation. Omicron breakthrough infection affords significantly increased de novo memory T cell responses to non-Spike viral antigens. Concerted T and B cell responses may provide durable and broad immunity.

## Abstract (web summary)

The SARS-CoV-2 Omicron variant possess many mutations within the receptor binding domain of the Spike protein, which confer increased transmissibility and higher antibody escape. Here, the authors carry out analysis of the serological and cellular immune responses of individuals with Omicron breakthrough infection.

## Introduction

On Nov 25, 2021, a new SARS-CoV-2 variant of concern (VOC), Omicron (B.1.1.529), was reported by South Africa^1,2^. Omicron has more than 30 mutations in the Spike protein, including 15 in the receptor-binding domain (RBD) that binds to the uptake receptor ACE2. These include shared mutations with the Alpha, Beta, Gamma, and Delta VOCs^2–5^. These previously well-studied mutations confer increased transmissibility^6,7^, higher viral binding affinity, and higher antibody escape^8–10^. Additional novel mutations are associated with immune evasion and antibody escape^5,11^, including a 40-fold reduced effect of IgG neutralizing antibodies in Pfizer BNT162b2–vaccinated patients as analyzed in Omicron VOC neutralization assays^12–14^. Omicron and its variants (BA1.1 and BA.2) have since been found worldwide despite the effectiveness of mRNA vaccines against Omicron^15,16^.

The super-spreader outbreak of Omicron VOC occurred at a Christmas party in Oslo Norway, on the 26 November 2021, with an index case who had recently traveled from South Africa. Participants were followed up and interviewed. 81 of the 110 (74%) developed Omicron SARS-CoV-2 breakthrough infection (BTI) with mild symptoms; 54% had fever^17^.

In contrast to data suggesting high levels of antibody evasion, bioinformatic analyses demonstrate that most immunodominant T cell epitopes are conserved^18^. We aimed to test if individuals with BTI had fully functional vaccine-generated T cell responses and to provide in-depth analyses of the T and B cell immune responses in Omicron BTI, see Supplementary Fig. 1 for overview of the study. 13 infected individuals were included for in-depth cellular analyses. Delta BTI (*n* = 13) and vaccinated health care workers (*n* = 14) served as controls. Cellular, serological and inflammatory immune responses were analyzed.

Here, we show that BTI in vaccinated individuals triggers robust vaccine recall responses of both T and B cell immunity including increases in activated SARS-CoV-2 Spike-specific cytotoxic T cells (CTL), activated follicular helper (T_FH_) cells, functional T cell responses, boosted humoral responses, and rapid release of Spike and RBD-specific IgG^+^ B cell plasmablasts and memory B cells into circulation. In addition, we find an emergent de novo CTL response to non-Spike antigens and an emergent anti-RBD plasma cell response that suggests adapted anti-Omicron B cell responses and concerted T and B cell immunity.

## Results

### Individuals with BTI and controls

Individuals with Omicron and Delta BTI (see Supplementary Fig. 2a) were recruited at similar time points after viral detection (median 11 and 10 days in Delta and Omicron BTI). The median duration of symptoms was similar (5 days) and the viral load was not significantly different (median ΔCt value was 24.9 for Delta BTI and 26.4 for Omicron BTI). Fully vaccinated healthy donors (HD) and COVID-19 convalescents bio-banked 3–6 months post-recovery from SARS-COV-2 Wuhan-Hu-1 (referred to as wild type, WT) infection served as controls (see Methods).

### Inflammatory markers

All individuals had a mild disease course, and none required hospitalization (Supplementary Fig. 2a). We found that the Omicron BTI group had an inflammatory signature compared to HD characterized by an increase of PF4 (CXCL4, released during platelet activation, *p* < 0.0001), MPO, GDF-15 and LBP (associated with granulocyte activation, tissue damage and monocyte activation; *p* < 0.0001, *p* = 0.0229, *p* < 0.0001 respectively), and an increased lymphocyte/monocyte ratio (Supplementary Fig. 2c–e).

The individuals with Delta BTI had a different plasma signature with additionally elevated CRP (*p* = 0.0132), Galectin-9 (linked to inflammation and tissue damage, *p* = 0.0004), and sCD14 (*p* = 0.0001, Supplementary Fig. 2f). In conclusion, we found a distinct inflammation during the acute phase of the breakthrough infection in Omicron and Delta BTI, although the findings were limited in comparison to that reported for severe COVID-19^19^.

### Analyses of Spike peptide-HLA multimer binding CD8^+^ cells

**Fig. 1:**
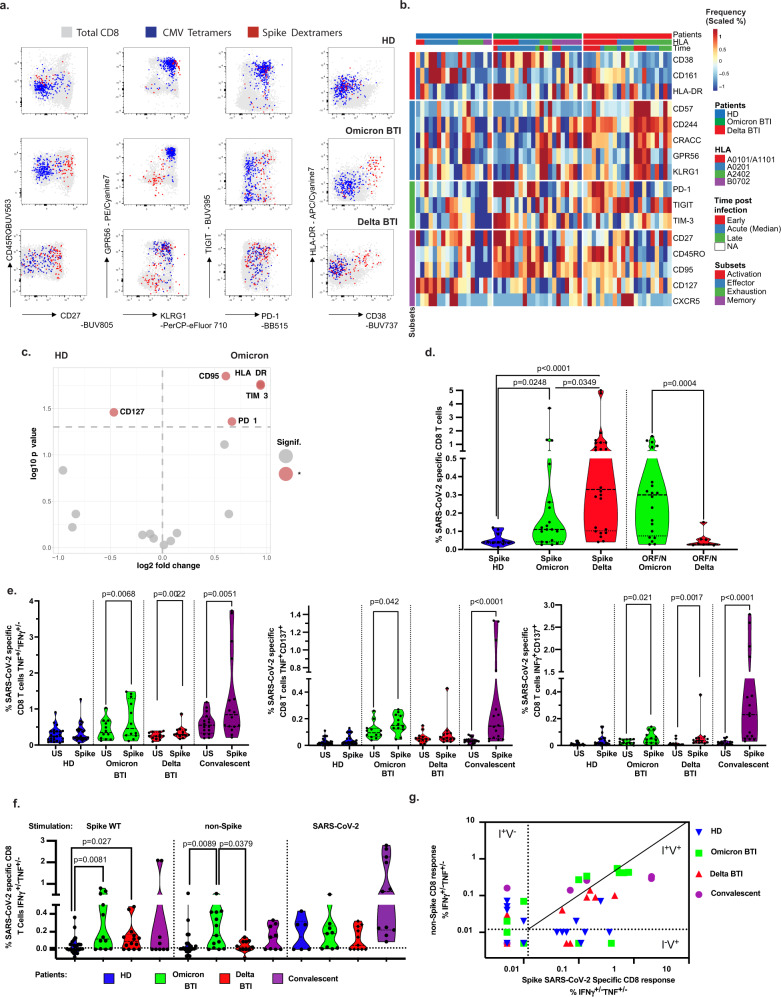
Cytotoxic cellular immunity during Omicron and Delta BTI. CD8^+^ T cells in Omicron or Delta BTI, vaccinated healthy donors (HD) or SARS-CoV2 WT convalescents were compared for their phenotypes (**a**–**d**) or functions in flow cytometry-based assays (**e**, **f**). **a** Characterization of Spike-, and CMV-specific CD8^+^ T cells. Anti-Spike Dextramer (red) and anti-CMV tetramer staining (blue) were combined with deep immune-phenotyping by flow cytometry and overlaid on total CD8 T cells (grey). Representative examples of single individual are shown. See Methods for peptides and peptide: HLA multimers. **b** Ex vivo immune phenotype of SARS-CoV-2 Spike-specific CD8^+^ T cells. A cold to hot heatmap represents the scaled frequency of each individual marker expressed by antigen-specific CD8^+^ T cells. Patient, HLA for A and B alleles, time post-infection (early acute <10 d, median acute for Day 10–11 or late acute >11 d) and marker subsets are indicated in the top three rows and in the leftmost column respectively. **c** Signature of Spike-specific CD8 T cells. The identification of specific markers is based on the fold change and significance in comparison to the phenotype of Spike-specific CD8 T cells from HD and is visualized by red dots on the volcano plot. **d** Quantification of SARS-CoV-2 Spike and non-Spike-specific CD8^+^ T cells defined by peptide: HLA multimers in Omicron and Delta BTI. Mann–Whitney test (two-sided) *P* values are shown. **e** Functionality of Spike-specific CD8^+^ T cells. PBMCs were stimulated overnight in vitro by overlapping SARS-CoV-2 Spike (wild type, WT) peptides or left unstimulated (US) and stained to assess the induction of activation markers. IFNγ and/or TNF responding cells or dual expression of IFN-γ, CD137 and TNF is shown. Wilcoxon test (two-sided) *P* values are shown for comparison between unstimulated and peptides stimulated samples. **f** Functionality of SARS-CoV-2 specific CD8^+^ T cells. Response over background of CD8^+^ T cells to the stimulation in vitro by Spike (WT) peptides, left; middle: pooled overlapping SARS-CoV-2 membrane (M) and nucleoprotein (N) peptides, and ORF peptides (See Methods and Table 2)—and right: the whole proteome of WT, 88 peptides from SARS-CoV-2 (S, M, N, E, O), see “Methods”. IFN-γ and/or TNF responding cells are shown. Mann–Whitney test *P* (two-sided) values are shown. **g** Biplot showing in vitro responses of CD8^+^ T cells to non-Spike peptide pools vs. Spike-peptides. Quadrants are labeled as described^28^: infected and nonvaccinated (I^+^V^−^), non-infected and vaccinated (I^−^V^+^); or infected and vaccinated (I^+^V^+^). See also Supplementary Figs. 3, 4. Source data are provided as a Source Data file.

To determine the activation of vaccine-induced T cell immunity, we analyzed CD8^+^ T cells specific for Spike peptides with MHC-class I restricted multimers (See Methods). We also performed deep immune-phenotyping on T cells to identify markers found in mild SARS-CoV-2 infection. High dimensionality analyses of Omicron BTI revealed that the majority of Spike-specific CD8^+^ T cells were activated expressing HLA-DR, CD38^20^, CD27 and PD-1^21^ (Fig. 1a, b and Supplementary Fig. 3a). In contrast, the Delta BTI group had a lower frequency of cells with this phenotype but cells more often expressed markers associated with terminal differentiation (KLRG1, GPR56)^22,23^, Fig. 1a, b and Supplementary Fig. 3. We also performed clustering (Supplementary Fig. 4a) that showed expression of activation/memory markers more frequently in Omicron BTI, while Delta BTI showed increased frequency of cells with effector profile^22–24^. Similarly, principal component analysis biplots showed that activation markers such as CD95, HLA-DR, PD-1^21^, and inhibitory receptor TIM-3 characterized the Omicron BTI response (vs. HD, Fig. 1c), while Delta BTI responses showed the influence of terminal effector molecules such as CD57, and CD244^25^ (Supplementary Fig. 3c). Both groups had a significant reduction of CD127, a marker found on quiescent SARS-CoV-2 -memory T cells in the steady state^26^ (Fig. 1b, c, Supplementary Fig. 3b, c).

The phenotype and frequency of T cells directed against cytomegalovirus (CMV) or Epstein–Barr virus (EBV) were largely unaltered during Omicron and Delta BTI (Fig. 1a and Supplementary Fig. 3d), therefore excluding systemic bystander activation^27^. It should be noted that each donor was screened for multimers restricted by different HLAs, and individuals may have multiple data points (potentially two for HLA-A*0101, -A*0201 or -A*2402 and one for HLA-B*0702). Patient alleles can be found in Supplementary Fig. 2a.

The frequency of Spike-specific CD8^+^ T cells in Omicron and Delta BTI was higher than in HD (*p* = 0.0248, and *p* < 0.0001, respectively), but was lower after infection with Omicron than with Delta BTI (*p* = 0.0349) (Fig. 1d, left).

### Analyses of non-Spike peptide-HLA multimer binding CD8^+^ cells

Next, we sought to evaluate whether we could detect immune responses directed against non-Spike proteins. We found a marked expansion of T cells that were stained by Class I restricted tetramers towards non-Spike peptides (ORF3a, ORF1ab and Nucleocapsid, see Methods), Fig. 1d, right. Moreover, Omicron BTI had increased frequency of non-Spike-specific CD8^+^ T cells compared to Delta BTI (*p* = 0.0004, Fig. 1d, right panel), and these had a memory phenotype, contrasting with an effector phenotype found in Delta BTI (Supplementary Figs. 3, 4b).

### Analyses of in vitro CD8^+^ T cell responses

As a corollary to the expanded and activated state of vaccine-specific CD8^+^ T cells, we found that individuals with Omicron BTI had significantly increased in vitro CD8^+^ T cell responses to Spike peptides, including significantly increased frequencies of cells secreting TNF or IFN-γ or both, TNF^+/−^IFN-γ^+/−^ (*p* = 0.0068), CD137^+^TNF^+^(*p* = 0.042), CD137^+^IFN-γ^+^ (*p* = 0.021) cells (Fig. 1e). Functional responses in SARS-CoV-2 specific CD8^+^ T cells were barely detectable in HD (3–4 months post-vaccine) but were still strong in convalescent patients (3–6 months post-recovery (*p* = 0.0051 for TNF^+/−^IFN-γ^+/−^ and *p* < 0.0001 for CD137^+^TNF^+^ or CD137^+^IFN-γ^+^, Fig. 1e). Spike-specific CD8^+^ T cell responses were significantly increased over HD in both Omicron and Delta BTI (*p* = 0.0083 and *p* = 0.0277 respectively), Fig. 1f.

We next analyzed in vitro CD8^+^ T cell responses to other (non-Spike) antigens of the SARS-CoV-2 virus. Omicron BTI and convalescent patients had detectable CD8 T cell response directed against Membrane-, Nucleoprotein- and ORF peptides (Fig. 1f, middle). Hence, strong vaccine-specific CD8^+^ T cell immunity to Spike peptides was accompanied by emergent immunity to SARS-CoV-2 epitopes that included a multitude of non-Spike epitopes, and this response was higher in Omicron than Delta BTI (*p* = 0.0379) and higher in Omicron BTI than in HD (*p* = 0.0098, Fig. 1f). Emergence of specific responses to Spike and non-Spike was visualized in a biplot of Spike vs. non-Spike response as recently described^28^. Here responses can be divided into quadrants: infected and nonvaccinated (I^+^V^−^), non-infected and vaccinated (I^−^V^+^); or infected and vaccinated (I^+^V^+^). About half the Omicron and Delta BTI were already found in the I^+^V^+^ quadrant (Fig. 1g). Some convalescent samples were also in this quadrant, but information on vaccination status was lacking on this group in our study.

We also found that CD8^+^ T cells from individuals with Delta BTI had increased cytotoxic properties compared to Omicron BTI in terms of granzyme-B response (*p* = 0.0173, Spike-specific) or perforin response (*p* = 0.0398, non-Spike-specific, Supplementary Fig. 4c). The augmented functionality was consistent with the terminal effector profile in Delta BTI seen in the phenotype analyses presented above (Fig. 1a, b, Supplementary Fig. 3a–c).

### Analyses of other T cell subsets and CD4^+^ T cells

**Fig. 2:**
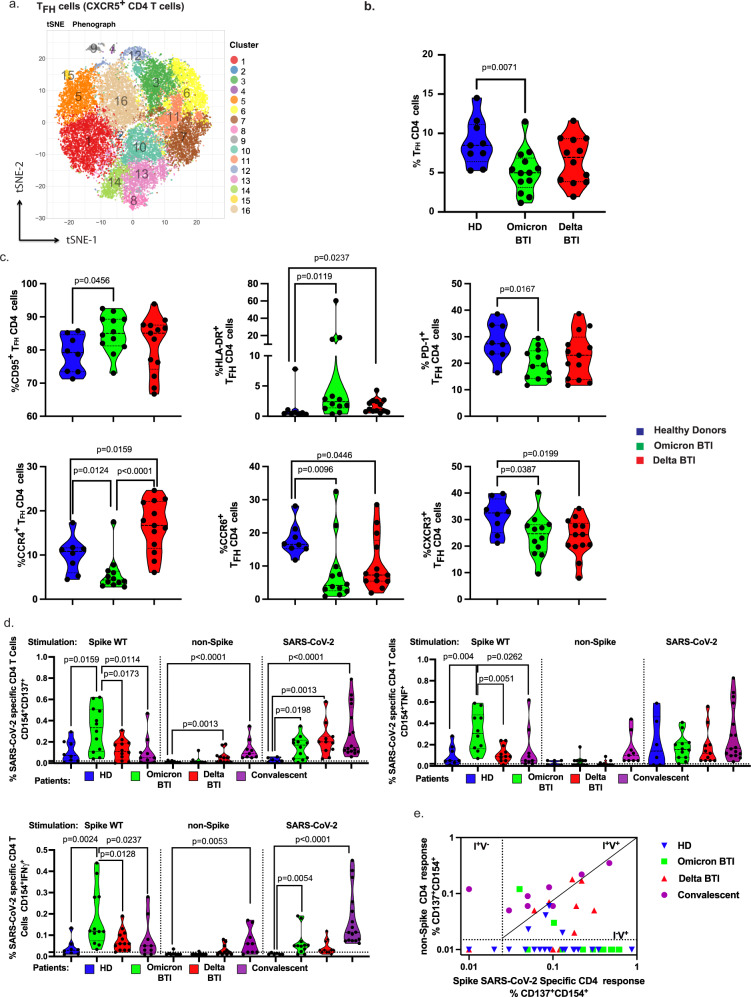
Th cellular immunity during Omicron and Delta BTI. Omicron or Delta BTI vaccinated healthy donors or SARS-CoV2 WT convalescents were analyzed to quantify their frequency and characterize the phenotypes of CD4^+^ T_FH_ cells (**a**–**c**) or to assess their functions (**e**, **f**). **a** Mass Cytometry profile of CXCR5^+^CD4^+^ Follicular helper T cells. Visualization by tSNE-plot of clusters automatically identified by Phenograph in total T_FH_ from concatenated files of vaccinated HD, Omicron BTI, and Delta BTI (see Methods and Supplementary Fig. 6 legend for details). **b** The absolute frequency of T_FH_ CD4 T cells in PBMCs for all BTI patients. **c** Frequency of T_FH_ cells expressing memory (CD95) or activation markers (HLA-DR), PD-1, and chemokine receptors (CCR4, CCR6, CXCR3). **d** Functional responses of SARS-CoV-2 specific CD4^+^ T cells. The specific responding CD4^+^ T cells are shown similarly as in Fig. 1f. **e** Biplot showing in vitro responses of CD4^+^ T cells to non-Spike peptide pools vs. Spike-peptides as described^28^: infected and nonvaccinated (I^+^V^−^), non-infected and vaccinated (I^−^V^+^); or infected and vaccinated (I^+^V^+^). Mann–Whitney test *P* values are shown for (**b**–**d**). See also Supplementary Fig. 6.

Among the other T cell subsets, we observed a peripheral decrease of TCRγ/δ^+^ T cells, and no significant modulation of MAIT cells or CD4/CD8 ratio (Supplementary Fig. 5). In the T helper (Th) subsets, we did not observe any general upregulation of markers associated with T cell differentiation, activation, exhaustion, or senescence. Next, we evaluated whether T follicular helper (T_FH_) cells were activated, this subset is involved in humoral responses and can be induced by SARS-CoV-2 mRNA vaccination^29^. An unbiased mass cytometry analysis identified T_FH_ cellular clusters in representative donors (Fig. 2a and Supplementary Fig. 6a, b, markers used for t-SNE analysis are described in Supplementary Fig. 4a). T_FH_ clusters which were found to be increased in Omicron BTI expressed PD-1 (important for B cell help, the germinal center reaction and plasma cell genesis^30^, cluster C3), proinflammatory marker such as CD161 (C7, C16) or activation markers such as CD38 or HLA-DR (C9), Fig. 2a and Supplementary Fig. 6. Finally, we extended our high-dimensional analysis to the entire cohort for all markers and performed supervised analysis. Mass cytometry (confirmed by flow cytometry staining) revealed that Omicron BTI was associated with a reduced frequency of T_FH_ cells (*p* = 0.0071, Fig. 2b) and an up-modulation of CD95 and HLA-DR (*p* = 0.0456 and *p* = 0.0119 respectively), Fig. 2c. By contrast there was a down-regulation of cells with chemokine receptors CXCR3 (*p* = 0.0387), CCR4 (*p* = 0.0124) and CCR6 (*p* = 0.0096), which are associated with T_FH_1, T_FH_2 and T_FH_17 responses, respectively^31^, Fig. 2c. The phenotype of T_FH_ in Delta BTI was similar with reduced T_FH1_, and T_FH17_ (*p* = 0.0199, *p* = 0.0446). However, T_FH2_ (CCR4^+^) cells were increased (*p* = 0.0159 vs HD and *p* < 0.0001 vs. Omicron BTI, Fig. 2). The impact of these T_FH_ profiles on isotype switching and the affinity maturation of B cells during BTI remains to be investigated.

### Analyses of in vitro CD4^+^ T cell responses

We next analyzed CD4^+^ Th cells for antigen-specific responses in vitro. Spike-specific Th responses were significantly increased in Omicron BTI compared to HD for CD154^+^CD137^+^, CD154^+^TNFα^+^ and CD154^+^IFNγ^+^ responses (*p* = 0.0159, *p* = 0.004, *p* = 0.0024, respectively, Fig. 2d and Supplementary Fig. 6e–g). The Th responses in Omicron BTI were also significantly stronger than responses observed in Delta BTI (*p* = 0.0173, *p* = 0.0051, *p* = 0.0128) and in convalescents (*p* = 0.0114, *p* = 0.0262, *p* = 0.0237). However, responses to non-spike proteins were limited and found only in Delta BTI where we detected significantly increased CD154^+^ CD137^+^ responses to non-Spike peptides (Fig. 2d, Supplementary Fig. 6g).

Responses to peptides covering the entire SARS-CoV-2 were more variable, but similar to those found against Spike peptides. Frequencies of CD154^+^CD137^+^ cells were significantly increased in Omicron and Delta BTI and convalescents vs HD (*p* = 0.0198, *p* = 0.0013 and *p* < 0.0001, Fig. 2d).

The Spike versus non-Spike responses for each patient in a biplot representation, also revealed that non-Spike responses had yet to appear in Omicron BTI, while some Delta BTI had already developed non-Spike responses (Fig. 2e).

### Analyses of B cell responses

**Fig. 3:**
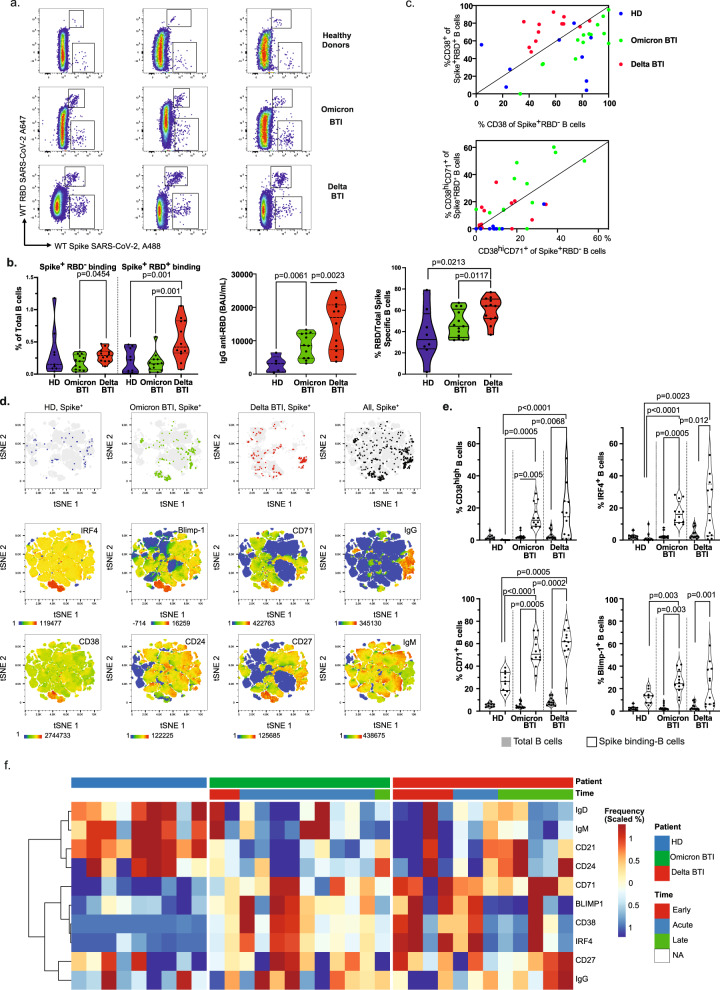
Humoral and B cell immunity during Omicron and Delta breakthrough infection. **a** Identification of B cells that bind Spike but not RBD (Spike^+^RBD^−^ B cells) or bind both Spike and RBD (Spike^+^RBD^+^ B cells) in total B cells from three individuals: Healthy Donor (HD), Omicron BTI and Delta BTI. **b** Quantification of Spike-binding and RBD-binding B cells. Left: Absolute frequency of Spike-binding and RBD-binding B cells. Middle: serum levels of anti-RBD IgG (BAU/ml). Right: Relative frequency of anti-RBD to spike-specific B cells. Ratio of RBD-binding B cells (see top region in a.)/total Spike-binding B cells is shown. **c** Biplot showing ex vivo profile of Spike^+^RBD^−^ and Spike^+^RBD^+^ B cells during BTI. Top: frequency of CD38 expression in Spike^+^RBD^−^ vs Spike^+^RBD^+^ B cells. Bottom: frequency of CD38^Hi^CD71^+^ plasmablasts in Spike^+^RBD^-^ vs Spike^+^RBD^+^ B cells. HD (blue), Omicron BTI (green) and Delta BTI (red) are shown. **d** Phenotype of Spike-binding B cells. Visualization by tSNE-plot of selected markers as indicated (bottom two rows) in total B cells and location of Spike-binding (Spike^+^) B cells (top row) in HD, Omicron BTI, Delta BTI and aggregated donors (20 000 cells from two individuals per group, total = 120 000 cells). Each marker is visualized by a cold to hot heatmap. **e** Quantification of anti-Spike antibody secreting cells (ASC). The frequency of specific markers for ASC among B cells (CD38, IRF4, CD71, BLIMP-1) are shown for Total B cells (blue) and Spike-binding B cells. **f** Characterization of Spike-binding B cells. A heat-plot shows the phenotype of Spike-specific B cells in the three groups. The normalized frequency of each marker is displayed and automatic hierarchical clustering of Spike-binding B cells for each patient is shown. Mann–Whitney test *P* values are shown for (**b**) and (**e**). See also Supplementary Fig. 7.

Next, we sought to investigate whether BTI could activate vaccine-B cell responses. Omicron BTI did not have increased number of B cells that bound Spike but not RBD (Spike^+^RBD^-^, Fig. 3a) or RBD-binding B cells (Spike^+^RBD^+^) compared to HD and had significantly lower frequencies than Delta BTI (*p* = 0.0454, *p* = 0.001 respectively, Fig. 3a, b). Spike^+^RBD^+^ B cells were increased in Delta BTI compared to HD (p = 0.001). However, IgG anti-RBD was significantly increased in Omicron BTI suggesting a boost of previous vaccine responses (*p* = 0.0061), but less so than in Delta BTI (*p* = 0.0023, Fig. 3a, b and Supplementary Fig. 7). To confirm the activation of the Spike^+^RBD^−^ and Spike^+^RBD^+^ B cells, we compared the expression of the activation marker CD38. In the Delta BTI, CD38 expression was higher in the RBD-binding Spike^+^RBD^+^ B cells than in the RBD^-^Spike^+^ B cells (10/12 were above the diagonal in Fig. 3c, upper panel). This was reversed in Omicron BTI where Spike^+^RBD^−^ B cells were more activated (0/13 were above the diagonal, Fig. 3c). However, when restricting the analysis to plasmablasts (CD38^Hi^CD71^+^ B cells^32–34^), we found a more balanced distribution with RBD-binding plasmablasts in the Omicron BTI biplot (7/13 were above the diagonal), Fig. 3c lower panel. Thus, anti-RBD plasmablast responses were elicited in Omicron BTI. Moreover, isotype switched Spike-binding B cells (not expressing IgM or IgD) were significantly more likely to express IgG in Omicron than in Delta BTI. This applied both for anti-RBD and anti-Spike (non-RBD), with *p* = 0.0062 and *p* = 0.0215 respectively, Supplementary Fig. 7. As expected, the concentration of anti-RBD IgG correlated significantly with the frequency of Spike-specific B cells (*p* = 0.0003) and increased significantly with time post-infection (*p* = 0.0004). At this early time-point, the concentration of anti-RBD IgA and the concentration of anti-Nucleocapsid IgG were still limited. The age of the participants was negatively associated with the humoral response in our small cohort (*p* = 0.0189), Supplementary Fig. 7.

We further quantified the B cell response by high dimensionality analyses. Omicron BTI had significantly increased Spike-specific plasmablasts (CD38^hi^CD27^hi^CD21^low^CD24^neg^CD71^hi^ IRF4^+^BLIMP-1^+^)^32–34^ where about half were IgG^+^ (Fig. 3c–f and Supplementary Fig. 7). The Spike-specific B cells also included activated IgG^+^CD24^+^CD21^+^ memory cells (CD27^+^CD71^+^, Fig. 3e, f, Supplementary Fig. 7a). Activated plasmablasts expressing CD38^Hi^, IRF4, or Blimp-1 and activated B cells/plasmablasts expressing CD71 were significantly overrepresented in Spike-binding B cells compared to the total B cell population in both Omicron and Delta BTI (*p* = 0.005 for all) and Delta BTI (*p* = 0.0068, *p* = 0.0132, *p* = 0.001 and *p* = 0.005, respectively, Fig. 3e). In conclusion, we found a surge of vaccine-specific differentiated plasmablasts and activated memory B cells in the Delta and Omicron BTI.

## Discussion

We here show that Omicron BTI is associated with a significant increase in frequencies of T cells specific for Spike SARS-CoV-2 peptide antigens derived from the vaccine. The T cell phenotype corresponded to T cell immunity in COVID-19 convalescents including activation markers and limited exhaustion markers. A more potent activation was seen in Omicron than in the Delta BTI where CD8^+^ T cell phenotypes were consolidated (in terms of effector markers) rather than activated. A corresponding increased cytotoxicity capacity in terms of effector functions (granzyme B and perforin) was found in Delta BTI. As a corollary, in vitro challenge with SARS-CoV-2 Spike peptides demonstrated a significantly increased CD8^+^ T cell responsiveness in Omicron BTI. We also found an increased frequency of CD8^+^ T cells specific for non-Spike peptides, and the magnitude of the response was higher in Omicron than in Delta BTI, suggesting the development of broad CD8^+^ T cell responses in Omicron BTI. T_FH_ cells, a specialized CD4^+^ Th cell subset able to induce affinity maturation of antibodies and development of memory B cells in germinal centers (GC), were decreased in the peripheral blood of Omicron and Delta BTI. However, they were significantly activated in Omicron BTI. We also found a significantly increased in vitro responsiveness of T helper cells to Spike peptides, and that this was even higher in Omicron than in Delta BTI. CD4 responses to non-Spike peptides were not induced in Omicron BTI but were significantly increased in Delta BTI (although lower than in convalescents).

Since Omicron VOC potentially escapes protective antibodies, exposed individuals will rely on the collaboration between vaccine-expanded T cell and B cell responses to counteract the Omicron infection. In silico^18^, in vivo^35^ and in vitro^36–41^ analyses suggest that Omicron mutations do not impact immunodominant Spike peptide antigens and that protective vaccine-generated T cell responses may be triggered by the Omicron infection. The current results confirm this perspective. However, the uncontrolled viral replication in a minority of vaccinated macaques after Omicron challenge correlated with limited humoral and cellular responses^35^. Importantly, in contrast to decaying antibody titres, SARS-CoV-1 T cell memory was long-lasting and specific T cell responses were still detected after 17 years^42^. The importance of T cells has been underlined in previous reports as T cells are necessary for rapid and efficient resolution of COVID-19^43,44^, for protection against severe infection in settings of low antibody levels, and for rapid viral control in the absence of antibodies - aborting infection in most but not all healthy individuals^45–47^.

The current results demonstrate an anamnestic humoral and cellular vaccine-elicited immunity in Omicron BTI including a CD8 T cell profile that resembled the more potent T cell activation found in infected, unvaccinated individuals^28,48^. This CD8 T cell profile contrasted with a terminal effector profile (phenotype and cytotoxic functions similar to vaccine boost responses^49,50^) found in Delta breakthrough infection. It is possible that the TCR affinity for peptides derived from VOC and/or the relative lack of neutralizing antibodies provide a greater antigenic challenge and activation of T cells in the Omicron BTI, thereby resembling the more potent T cell activation found in unvaccinated individuals^28,48^, while less activated responses in Delta BTI may reflect more effective antibodies, lower antigenic challenge or resemble responses found for particular SARS-CoV-2 peptides in COVID-19^51^. The re-challenge may have implications for durability of the T cell responses.

Our study analyzed emerging responses at median of 10–11 days after viral detection in Omicron and Delta BTI. The heterogeneity in responses may relate to escape from antibody protection^2,4–11^, the specific dynamic of viral replication for each VOC, or pre-existing heterogeneous immunity elicited by the vaccines^28^. Moreover, some responses may vary with time post-infection, and may require longitudinal follow-up. In this regard, the circulating T_FH_ cell frequencies were reduced in comparison to vaccinated non-infected HD for both Omicron and Delta BTI during the acute phase of BTI, including CXCR3^+^ T_FH1_ and CCR6^+^ T_FH17_^31^, suggesting that T_FH_ might still be sequestered in lymphoid tissues (GC in lymph nodes). The dynamic and the phenotype of circulating T_FH_ and GC T_FH_ during and after acute SARS-CoV-2 infection has been documented, and differed from the response to vaccination (including vaccinated convalescent patients), see^31^ for review. A further longitudinal study of circulating T_FH_ is required for Spike and non-Spike-specific cells in order to evaluate whether circulating T_FH_ can be considered as a biomarker for long-lasting and protective humoral immunity.

The T cell responses were accompanied by a significantly boosted IgG anti-RBD response and a surge of IgG^+^ RBD and Spike-specific B cells that were plasma cell precursors or activated memory B cells. B cell responses in Omicron BTI favored non-RBD-Spike-binding B cells, but emergent plasmablasts showed a balanced anti-RBD and anti-Spike (non-RBD) response demonstrating an evolving anti-RBD response in Omicron BTI. One explanation of this difference may be the loss of affinity for Omicron RBD^4,5,8,12^ in vaccine-generated RBD-binding B cells and the requirement for affinity maturation or de novo responses to generate neutralizing antibodies. Nevertheless, we found that plasmablasts in Omicron BTI had a more balanced Spike^+^RBD^+^/Spike^+^RBD^-^ ratio, suggesting the development of a new serological RBD-response. This was supported by significantly increased IgG anti-RBD levels in Omicron BTI. In line with this, a recent study showed that 30% of RBD WT-binding memory B cells in recipients of two doses of the BNT162b2 mRNA vaccine could be activated in vitro to secrete Omicron neutralizing IgG, suggesting broadly cross-reactive B cells^52^. Systematic study of emergent B cells including VDJ/VJ clonal evolution analyses of B cells with variant-specific and cross-reactive RBD-binding B cells are suggested.

In the current study, as many as 74% of the participants developed an Omicron BTI despite being fully vaccinated. Preliminary analyses^12^ suggested that the increased transmission and breakthrough infections relate to escape of antibody neutralization as sera from Pfizer BNT162b2–vaccinated individuals showed dramatically reduced viral neutralization. In addition, neutralizing antibodies (NAbs) peak and decay with time after vaccination.^53^ Most infected individuals at the Christmas party were young to mid-aged adults that had been vaccinated 3-6 months before the party, suggesting some decay of NAb titers.

mRNA-based vaccination induces a robust but transient SARS-CoV-2-specific antibody response, and persistent SARS-CoV-2-specific GC B cell^54–56^ and T_FH_ cell response^57,58^. The magnitude and the breadth of B cell response directed against SARS-CoV-2 Spike epitopes are also modulated by the number of vaccine doses. After two doses responses towards the membrane-proximal S2 region were dominant, while increased responses against the membrane distal S1 region including the N-terminal domain (NTD) and RBD were seen after third challenge^55,59^. Moreover, vaccination generates responses that include the development of Spike-specific Th cells and class switched memory B cells that cross-react with Alpha, Beta, and Delta RBDs, and are capable of rapidly producing neutralizing antibodies after stimulation^55,59^. It should be noted that we probed with Wuhan-Hu-1 RBD, and that cross reactivity may be especially limited in Omicron BTI^4,5,8,12,55^. Thus, a de novo response may be required for the development of optimal NAb that bind Omicron RBD.

We found a boosted humoral and cellular vaccine-elicited immunity and a de novo memory CTL cell responses to non-Spike viral antigens in Omicron BTI. The emergent anti-RBD plasma cell responses suggested adapted anti-Omicron B cell responses and concerted T and B cell immunity. Further longitudinal follow-up of the immunity after breakthrough infection is required including monitoring of cell subsets, Omicron specific B cells and Omicron Neutralizing Ab, and the degree of long-term protection from re-infection with new variants. Similar studies are also needed in patients that have poor vaccine responses. Better understanding of evolving immunity and durability of responses will be important for the design of next-generation vaccines.

## Methods

### Omicron and Delta BTI and controls

The current work is compliant with the current ethical and judicial framework regulating research on human subjects. Ethical approvals were obtained from regulatory committee authority (Approval numbers: REK 2021.233704; REK 2020.135924; REK 2021.229359). Donors signed informed consent forms.

We included four main cohorts: (1) participants from the Christmas party with Omicron BTI (*n* = 13, mild disease) as described^17^, (2) donors with Delta BTI (*n* = 13, mild disease) from the Norwegian Institute of Public Health (NIPH) Young Adult cohort (see Table 1 and Supplementary Fig. 2a for overview), (3) control fully vaccinated health care workers (healthy donors, labeled HD) at the Oslo University Hospital (*n* = 14), and (4) non-hospitalized SARS-CoV-2 (Wuhan-Hu-1)-infected convalescent blood bank donors (labeled convalescent WT) who were bio-banked 3–6 months post-recovery in August 2020–July 2021 and served as positive controls (*n* = 16).

**Table 1:** Overview of BTI.

	Delta BTI (*n* = 13)	Omicron BTI (*n* = 13)^a^	Non-infected, vaccinated health care workers (HD) (*n* = 14)
*Age (y)*			
Mean (range)	27 (21–30)	37.3 (28–50)	31.9 (25–44)
Median	27	38	31
*Sex*			
Female, *n* (%)	8 (61.5)	9 (69.2)	10 (71.4)
Male, *n* (%)	5 (38.5)	4 (30.8)	4 (28.6)
*Vaccine type, dose 2*			
BNT162b2 (Pfizer-BioNTech), *n* (%)	8 (61.5)	8 (61.5)	14
mRNA-1273 (Moderna), *n* (%)	5 (38.5)	5 (38.5)	0
*Time since infection (d)*			
Mean (range)	12.3 (3–22)	9.9 (6–13)	–
Median	11	10	–
*Time since dose 2 vaccination (d)*			
Mean (range)	147 (92–297)	117 (94–171)	115 (92–167)
Median (range)	115	112	115
*Time between dose 1 and 2 (d)*			
Mean (range)	49 (27–83)	46 (36–66)	68 (64–87)
Median	46	43	64
*Vaccine type, dose 1*			
BNT162b2 (Pfizer-BioNTech), *n* (%)	9 (69.2)	8 (61.5)	14
mRNA-1273 (Moderna) *n* (%)	4 (28.6)	4 (30.8)	0
*PreviousCOVID-19*			
*n* (%)	0	1 (7.7)	0

^a^One of the Young Adult cohort was found to be infected with the Omicron variant and was subsequently included with the Omicron BTI.

The Omicron BTI were collected as part of the routine outbreak investigation performed by the Norwegian Institute of Public Health (NIPH). Subjects with Delta BTI were recruited from the Young Adult Cohort, NIPH. This cohort was established at NIPH and designed to understand the consequences of the pandemic among subjects aged 18–30 years. Blood samples (PBMC, serum, plasma) were collected. SARS-CoV-2 RNA in nasal/pharyngeal swabs was whole-genome sequenced or typed by variant-specific SNP PCR to confirm infection by the Omicron or Delta VOC (as designated by the World Health Organization). One of the intended 14 Delta BTI controls was disqualified as a control since sequencing revealed infection by Omicron and not Delta VOC. This sample was transferred to the Omicron BTI for a total of *n* = 13.

All participants had received both doses of SARS-CoV-2 vaccines (either BNT162 or mRNA-1273) according to the Norwegian National Vaccination Program. The two vaccines were given with an interval of 4-12 weeks (median of 6.5 weeks). Subjects had received their second vaccine doses between 13 and 42 weeks prior to this outbreak (with a median of 16 weeks and mean of 21 weeks).

Information on vaccination and infection were obtained from the Norwegian Immunization Registry (SYSVAK) and Norwegian Surveillance System for Communicable Diseases (MSIS).

### Sample preparation and HLA typing

All participants were screening for HLA typing using the freshly isolated PMBC samples. Cells were stained with anti- HLA-A2 (Biolegend), HLA-A24 (LSBio) and HLA-B07 (Biolegend) and typed by flow cytometry. Frozen PBMCs from individuals positive for these HLAs were subsequently stained with the corresponding Dextramers/ Tetramers Class I restricted (HLA-A*02:01, HLA-A*24:02, and HLA-B*07:02 respectively). Negative patients for one of the HLA-A screened were directly tested for HLA-A*01:01, HLA-A*11:01 by Dextramer staining directed against SARS-CoV-2 and control virus (CMV, EBV, Influenza, see the overview of peptides below in the section on specific memory CD8 T cells). Sample within the groups had similar HLA distribution with HLA A02 and A24 in >5/13 BTI, and B07 in more than 3 BTI.

Each sample consisted of 2 frozen aliquots with an average cell number of 10 million cells and viability above 95% per donor. Samples were thawed at 37 °C and immediately transferred into a complete RPMI medium (10% FCS, 1% penicillin /streptomycin, glutamine, 10 mM HEPES). After the first wash, thawed cells were incubated during 15 min at room temperature with DNAse (STEMCELL). Live cells were purified by removing dead cells using a column-based magnetic depletion approach according to the manufacturer’s recommendations (Miltenyi). Vaccinated healthy donor PBMCs matched for at least one of the donor HLA alleles were included in each experiment as control for specific T cell identification. VeriCells were included in each experiment as control for phenotypic markers.

### Flow cytometry

Thawed peripheral blood mononuclear cells (PBMCs) were stained with antibody panels to quantify and phenotype-specific T cell responses to Spike peptides (two million cells per sample) and B cell responses to RBD or Spike protein (one million cells per sample). Cells were acquired on a BD FACSymphony (BD Biosciences) or Attune NxT (ThermoFisher). The gating strategy can be found in Supplementary Fig. 8.

The following mAbs and stains were utilized for acquisition on BD FACSymphony: BB515 Mouse Anti-Human CD279 (PD-1) Clone EH12.1, BD Biosciences, PerCP-eFluor 710, KLRG1 Monoclonal Antibody (13F12F2), eBioscience, PE/Cyanine7 anti-human GPR56, Clone CG4, Nordic Biosite, Alexa Fluor 700 anti-human CD244 (2B4), clone C1.7, Nordic Biosite, APC/Cyanine7 anti-human HLA-DR, clone L243, Nordic Biosite, BV480 Rat Anti-Human CXCR5 (CD185) (Clone: RF8B2) BD Biosciences, BB515 Mouse Anti-Human CD38, clone, HIT2 BD Biosciences, Brilliant Violet 570™ anti-human CD3, Nordic Biosite, Brilliant Violet 605, CD127 Mouse anti Human, Clone HIL 7R M21, BD Biosciences, Brilliant Violet 650, CD161 Mouse anti Human, clone: DX12, BD Biosciences, BV711 Mouse Anti-Human TIM-3 (CD366), clone 7D3, BD Biosciences, BV750 Mouse Anti-Human CD8, clone SK1, BD Biosciences, Brilliant Violet 785 anti-human CD57 Recombinant, clone QA17A04, Nordic Biosite, BV421 Mouse Anti-Human CD319 (CRACC), BD Biosciences, BUV395 Mouse Anti-Human TIGIT, clone 741182, BD Biosciences, Live/dead Fixable Blue Dead Cell Stain Kit, for UV excitation, Thermo Fisher Scientific, BUV563 Mouse Anti-Human CD45RO, clone UCHL1, BD Biosciences, BUV615 Mouse Anti-Human CD95, clone DX, BD Biosciences, BUV661 Mouse Anti-Human CD4, clone SK3, BD Biosciences, BUV737 Mouse Anti-Human CD38, clone HB7, BD Biosciences, BUV805 Mouse Anti-Human CD27, clone L128, BD Biosciences. VeriCells PBMC (BioLegend) were included as controls.

Frequency values were calculated based on the percentage of the parent immune cell population and phenotypic markers were gated individually for each sample and calculated as % of positive cells. High-dimensional phenotypic profiles and sample distributions were shown using uniform manifold approximation and projection. Data analysis was performed using CYTOGRAPHER® (ImmunoScape cloud-based analytical software), custom R-scripts, GraphPad Prism (GraphPad Software) and FlowJo v10 software (BD Life Sciences). Statistical significance was set at a threshold of *p < 0.05, **p < 0.01, and ***p < 0.001.

### In vitro stimulation assays

Thawed cells were stimulated for 16 h with SARS-CoV-2 PepTivator Spike protein peptides consisting of 15-mer sequences with 11 amino acid overlaps (Wuhan-Hu-1, i.e. wild type WT. Miltenyi Biotec). For non-Spike (WT) responses cells were stimulated with Nucleoprotein (PepTivator SARS-CoV-2 Prot N) and Membrane protein (PepTivator SARS-CoV-2 Prot M), consisting of 15-mer sequences with 11 amino acid overlaps in addition to the 4 ORF1ab/Orf3a peptides in Table 2, i.e. stimulated with M + N + O, Fig. 1f (middle). Alternatively, in Fig. 1f (right), cells were stimulated with 88 pooled WT immunodominant oligopeptides from the whole proteome (PepTivator SARS-CoV-2 Select, Miltenyi Biotec) consisting of peptides from structural proteins (S, M, N, E) as well as non-structural proteins (O). Peptide stimulation was performed on 1 million PBMCs per condition in the presence of costimulatory antibodies against CD28 and CD49d (BD Biosciences) and Brefeldin-A (10 μg/mL, Millipore Sigma). SARS-CoV-2-specific T cells were identified by dual expression of CD40L (CD154) and CD137, interferon-gamma (IFN-γ), interleukin-2 (IL-2), or tumor necrosis factor (TNF) for CD4^+^ T cells and by dual expression of IFN-γ and TNF or CD137 and IL-2, TNF or IFN-γ for CD8^+^ T cells.

**Table 2:** Peptide: HLA multimers.

	HLA-A0201	HLA-A2402	HLA-B702	HLA-A0101	HLA-A1101
Spike	YLQPRTFLL	QYIKWPWYI	SPRRARSVA	LTDEMIAQY	
	RLNEVAKNL	NYNYLYRLF	APHGVVFL	YTNSFTRGVY	
	LITGRLQSL				
Non-Spike	LLYDANYFL ORF3a 139-147	VYFLQSINF ORF3a 114-122	SPRWYFYYL Nucleocapsid 105-113	FTSDYYQLY ORF3a 207-215 and TTDPSFLGRY ORF1ab 1637-1646	KTFPPTEPK Nucleocapsid 362-370
CMV	NLVPMVATV	QYDPVAALF	RPHERNGFTVL	VTEHDTLLY	
EBV	FLYALALLL		RPPIFIRRL		
FLU				CTELKLSDY	

### Detection of specific memory CD8 T cells

Antigen-specific CD8 T cells were detected by peptide: HLA multimers (see Table 2 for overview). The peptides listed below are referenced individually in the Supplementary Section. Spike-Specific CTL were detected using PE-conjugated Dextramers (Immudex) targeting Spike and restricted to HLA-A*0101 (LTDEMIAQY), HLA-A*0201 (YLQPRTFLL), HLA-A*2402 (QYIKWPWYI), and HLA-B*0702 (SPRRARSVA). The panel was expanded using Flex-T tetramer according to the manufacturer´s instructions (BioLegend). We UV-exchanged peptides for Spike epitopes restricted to HLA-A*0101 (YTNSFTRGVY), HLA-A*0201 (LITGRLQSL and RLNEVAKNL), HLA-A*2402 (NYNYLYRLF), and HLA-B*0702 (APHGVVFL) and tetramerized with Streptavidin-PE (Biolegend). A similar approach was performed for non-Spike derived epitopes, including HLA-A*0101 (ORF3a, FTSDYYQLY and ORF1ab, TTDPSFLGRY), HLA-A*0201 (ORF3a, LLYDANYFL), HLA-A*2402 (ORF3a, VYFLQSINF), and HLA-B*0702 (Nucleoprotein, SPRWYFYYL) and tetramerized with Streptavidin-APC (Biolegend). CMV-and EBV/FLU-specific CD8 T cells were generated similarly and tetramerized using Streptavidin-PECF594 (Biolegend) and Streptavidin-PE-Cy5 respectively. CMV-derived epitopes were for HLA-A*0101 (DNA polymerase processivity factor, VTEHDTLLY), HLA-A*0201 (65 kDa phosphoprotein, NLVPMVATV), HLA-A*2402 (65 kDa phosphoprotein, QYDPVAALF), and HLA-B*0702 (65 kDa phosphoprotein, RPHERNGFTVL) and EBV derived epitopes were for HLA-A*0201 (EBV LMP2, FLYALALLL) and HLA-B*0702 (EBV antigen 3, RPPIFIRRL). A Flu peptide was for HLA-A*0101 (Nucleoprotein, Influenza A virus CTELKLSDY). All peptides were ordered from Genscript with a purity above 85% by HPLC purification and mass spectrometry. Lyophilized peptides were reconstituted at a stock concentration of 10 mM in DMSO.

Antigen-specific multimer CD8 T cells were identified by fine manual gating, examples of stained cells for peptide:HLA multimers as listed in Table 2 can be seen in Supplementary Fig. 9. The designation of bona fide antigen-specific T cells was further dependent on (a) the detection cut-off threshold (≥5 events to be detected), (b) the background noise (frequencies of specific CD8^+^ T cells must be greater than frequencies from the corresponding CD4^+^ T cell population) as unbiased objective criteria for antigen-specificity assessment. Spike and non-Spike Dextramers staining have been extensively validated in COVID-19 convalescent patients and in SARS-CoV-2 vaccinated healthy donors during longitudinal follow-up.

### Mass cytometry

#### Antibody staining panel setup

Purified CD19 (Biolegend), TCRVα7.2, CD160 and KLRG1 (R&D Systems) antibodies lacking carrier proteins (100 μg/antibody) were conjugated to DN3 MAXPAR chelating polymers loaded with heavy metal isotopes following the recommended labeling procedure (Fluidigm). The rest of the following mAbs were directly purchased from Fluidigm: y89 anti-CD45, 106Cd anti-CD45, 110Cd anti-CD45, 111Cd anti-CD19, 112Cd anti-CD45, 114Cd anti-CD45, 116Cd anti-CD45, 141Pr anti-CCR6, 142Nd anti-CD57, 143Nd anti-CD45RA, 144Nd anti-CD38, 145Nd anti-CD4, 146Nd anti-CD8, 147Sm anti-CD20, 148Nd anti-CD14, 149Sm anti-CD25, 150Nd anti-TCRVα7.2, 151Eu anti-lambda, 152Sm anti-TCRγδ, 153Eu anti-TIM-3, 154Sm anti-CD3, 155Gd anti-CD27, 156Gd anti-CXCR3, 158Gd100 anti-CCR4, 159Tb anti-TIGIT, 160Gd anti-Kappa, 161Dy anti-CD160, 162Dy anti-CD95, 163Dy anti-CRTH2, 164Dy anti-CD161, 165Ho anti-CD127, 166Er anti-CD85j, 167Er anti-CCR7, 168Er anti-CD71, 169Tm anti-NKG2A, 170Er anti-HLA-DR, 171Yb anti-CXCR5, 172Yb anti-KLRG1, 173Yb anti-CD141, 174Yb anti-PD anti-1/CD279, 176Yb anti-CD56, 209Bi anti-CD16. All labeled antibodies were titrated and tested by assessing relative marker expression intensities on relevant immune cell subsets in commercial lyophilized PBMCs from healthy donors (VeriCells, BioLegend). Antibody mixtures were prepared freshly and filtered using a 0.1 mM filter (Millipore) before staining.

#### Sample staining and acquisition

Cryo-preserved PBMCs were enriched for live cells by magnetic depletion of dead cells (Dead cells removal microbeads, Myltenyi) in presence of Citrate buffer. One Million cells per donor samples, healthy donor PBMCs and VeriCells were seeded in 96-well plate. Cells were washed, and each well was then stained with 100 μL of a unique double metal-labeled (Y89, Cd-106, Cd-110, Cd-112, Cd-116 and Cd-196) anti-CD45 antibody mix to further barcode the cells of individual donor. Cells were then washed twice, and five samples were combined into a single well. Cells were first stained with Fc block (BD Biosciences) during 10 min at room temperature. Cells were then washed and stained with the heavy metal-labeled antibody mixtures for 30 min on ice and 200 μM cisplatin during the last 5 min for the discrimination of live and dead cells. Cells were washed twice and fixed in 2% paraformaldehyde in PBS overnight at 4 °C. Cells were then washed and resuspended in 250 nM iridium DNA intercalator (Fluidigm) in 2% paraformaldehyde/PBS at room temperature. Cells were washed, pooled together, and adjusted to 0.5 million cells per milliliter in MaxPar water together with 10 % equilibration beads (EQ Four element calibration beads, Fluidigm) for acquisition at 250 events/ second on a HELIOS mass cytometer (CyTOF, Fluidigm).

#### Data analysis

After mass cytometry acquisition, signals for each parameter were normalized based on EQ beads (Fluidigm). Each sample was manually de-barcoded followed by gating on live CXCR5^+^CD4^+^ T cells for T_FH_ analysis (CD45^+^ DNA^+^ cisplatin^–^ CD3^+^ cells) after gating out residual antigen-presenting cells (HLA-DR^+^CD3^−^ such as monocytes (CD14) and B cells (CD19) using FlowJo (Tree Star) software.

### Detection of SARS-CoV-2 specific memory B cells

Spike-specific B cells were detected using either sequential staining of biotinylated Recombinant SARS-CoV-2 Spike-Trimer (HEK) (Miltenyi) combined with streptavidin-PE or with probes already conjugated with Alexa Fluor 647 for Spike RBD (R&D Systems) and conjugated with Alexa Fluor 488 for Full-length spike protein (R&D Systems, adapted from reference^60^). 2 × 10^6^ of cryo-preserved PBMC samples were transferred in a 96-well U-bottom plate. Cells were first stained with Fc block (BD Biosciences) for 15 min at room temperature. Cells were then washed and stained separately with either 100 ng of Spike Trimer alone or probe master mix containing 200 ng spike-A488, and 25 ng RBD-A647 for 1 h at 4 C. Following incubation with antigen probes, cells were washed twice and stained with Blue Live Dead (Thermofischer) during 10 min at room temperature. Cells were washed again and stained with antibodies according to manufacturer protocols: BUV805-Mouse Anti-Human CD7, clone M-T701 BD Biosciences, BUV805-Mouse Anti-Human CD14, clone M5E2, BD Biosciences, BV711-Mouse Anti-Human CD19, clone HIB19, BD Biosciences, BUV395-Mouse Anti-Human CD20, clone 2H7, BD Biosciences, BUV737-Mouse Anti-Human CD21, clone B-ly4, BD Biosciences, BUV615-Mouse Anti-Human CD24, clone ML5, BD Biosciences, BV786-Mouse Anti-Human CD27, clone L128, BD Biosciences, BB515-Mouse Anti-Human CD38, clone HIT2, BD Biosciences, PecyPE-Cy-7-Mouse Anti-Human CD71, clone CY1G4, BD Biosciences, BV605-Mouse Anti-Human IgD, clone IA6-2, BD Biosciences, Percp.5PerCP-Cy5.5 Mouse Anti-Human IgM, clone MHM-88, Biolegend, BV421-Mouse Anti-Human IgG, clone G18-145, BD Biosciences, APC-H7-Mouse Anti-Human HLA-DR, clone L243, Biolegend, and BV480-Rat Anti-Human CXCR5, clone RF8B2, BD Biosciences for 30 min on ice. Cells stained with the Spike Trimer were fixed with the transcription factor buffer (Thermofischer) and intra-cellularly stained for IRF4 (eFluor660, clone 3E4, Thermofischer) and Blimp-1 (PE-CF594, clone 6D3, BD Biosciences). Cells stained with RBD and full Spike were fixed overnight in 1% PFA. Samples were acquired on BD FACSymphony.

### Inflammatory markers

The following enzyme-linked immunosorbent assay (ELISA) kits were used according to manufacturer protocols. From R&D Systems: Human CD14 DuoSet ELISA (DY383), Human CD163 DuoSet ELISA (DY1607), Human LBP DuoSet ELISA (DY870-05), Human Galectin-9 DuoSet ELISA (DY2045), Human GDF-15 Quantikine ELISA Kit (DGD150), Human CXCL4/PF4 Quantikine ELISA (Kit DPF40), Human IFN-alpha (41100); from Ebioscience: Human MPO Instant ELISA Kit (BMS2038INST); from Thermo Scientific: Invitrogen novex IP 10 Human ELISA Kit (KAC2361); from Abcam Human C-Reactive Protein/CRP (Ab99995); from MyBioSource: Human zonulin ELISA Kit (MBS706368); from Meso Scale diagnostics: human Calprotectin (F21YB-3). Acute phase reactants were analyzed by the Department of Medical Biochemistry at the Oslo University Hospital as described^61^.

### Serology

A multiplexed bead-based flow cytometry assay, referred to as microsphere affinity proteomics (MAP), was adapted for detection of SARS-CoV-2 and the receptor-binding domain (RBD) antibodies as described^62–65^.

### Statistics

Comparative analyses of frequencies of cell subsets and marker expression are presented by GraphPad Prism version 9 with violin plots and dashed lines to indicate median and interquartile range showing all data points, and the difference between control and test group was tested using Mann–Whitney U test for unpaired data and Wilcoxon test on paired samples for the comparison between unstimulated and peptides stimulated samples. Tests were two-sided. Values of *p* < 0.05 were considered statistically significant. Correlations were calculated with Pearson’s test. A correlation matrix was calculated comparing phenotypic and serological marker variables in a pairwise fashion, using the corr.test function from the psych CRAN package; the corrplot package was subsequently used to graphically display the correlation matrix. Resulting *P* values were adjusted for multiple testing using the Bonferroni method. Pearson’s correlation coefficients were indicated by a heat scale whereby red color shows positive linear correlation, and blue color shows negative linear correlation. The volcano plots and the correlation matrix were integrated as package in CYTOGRAPHER^®^, ImmunoScape cloud based analytical software.

### Reporting summary

Further information on research design is available in the Nature Research Reporting Summary linked to this article.

## Supplementary information

Supplementary Data

Reporting Summary

## Data Availability

Source data are provided with this paper. The datasets generated and analyzed during the current study are also available from the corresponding author on reasonable request. The patient samples are not available on request due to restricting ethical and legal approvals.

## Source data

Source data
