# Supplementary material for: Immune responses in Omicron SARS-CoV-2 breakthrough infection in vaccinated adults
Source: Nat Commun. 2022 Jul 18;13:4165. doi: 10.1038/s41467-022-31888-y (PMC9293966; doi:10.1038/s41467-022-31888-y)
Supplement: Supplementary file 1 — Supplementary Data [file 41467_2022_31888_MOESM1_ESM.pdf]

Kared H., et al *Immune responses in Omicron SARS-CoV-2 breakthrough infection in vaccinated adults*

Inventory of Supplementary Information

Supplementary Figures, Methods and References

Supplementary Figures and tables

Supplementary Figure 1. Schematic overview of study design. This Figure relates to Supplementary Figure 2 and Table 1 (overview of the individuals in the groups)

Supplementary Figure 2. Symptoms and Inflammation during Omicron and Delta breakthrough infection. This Figure presents characteristics of individuals with Omicron and Delta BTI and inflammatory markers. Relates to Table 1 and Supplementary Fig. 1.

Supplementary Figure 3. $CD8^+$ T cell immunity during Omicron and Delta BTI. This Figure provides further detail on Spike specific and non-spike specific CD8+ T cells with PCA biplots, clustering and volcano plots that add to Figure 1 and also relate to Supplementary Figure 4 and 5.

Supplementary Figure 4. $CD8^+$ T cells analysis of granzyme B and perforin. This Figure provides hierarchical clustering of phenotypes of Spike specific and non-spike specific CD8+ T cells, relating to Figure 1 and Supplementary Figure 3. It also provides functional analysis of granzyme and perforin secretion, extending the results from the phenotype analyses (Figure 1 and Supplementary Figure 3).

Supplementary Figure 5. Non-classical T cells and CD4/CD8 ratio during Omicron and Delta BTI. This Figure shows innate T cells and CD4/CD8 ratio and completes the T cell subtypes before the $CD4^+$ T cell subset is analyzed. Relates to the T cell Figures 1&2 and Supplementary Figures 2-4.

Supplementary Figure 6. $CD4^+$ T helper cell immunity during Omicron and Delta BTI. This Figure provides further data on $CD4^+$ T helper cell immunity and adds to Figure 2.

Supplementary Figure 7: B cell and humoral immunity during Omicron and Delta BTI. This Figure provides further details on the B cell analyses presented in Figure 3.

Supplementary Figure 8: Gating strategy. This Figure presents the gating strategy for the analyses of the cell subsets in the study.

Supplementary Methods and Supplementary References

This section lists peptides used in the study and provides references to previous studies of these immunodominant peptides.

Supplementary Figures

Supplementary Fig. 1. Schematic overview of study design
Samples were collected from the four cohorts: participants from the Christmas party with Omicron BTI, donors with Delta BTI (see Table 1 and Supplementary Figure 2a for overview), control fully vaccinated health care workers (healthy donors, labelled HD) at the Oslo University Hospital, and non-hospitalized SARS-CoV-2 (Wuhan-Hu-1)-infected convalescent blood bank donors. SARS-CoV-2 RNA in nasal/pharyngeal swabs was whole-genome sequenced or typed by variant specific SNP PCR to confirm infection by the Omicron or Delta VOC and for estimation of viral load. Blood was collected for: analyses of plasma/serum soluble mediators (inflammatory and acute phase markers and cytokines), left; analyses of humoral antibody responses as well as Spike binding B cells (middle), and further immune cell composition including T cell response assays (right). The Figure was generated with licensed BioRender software.

Supplementary Figure 1

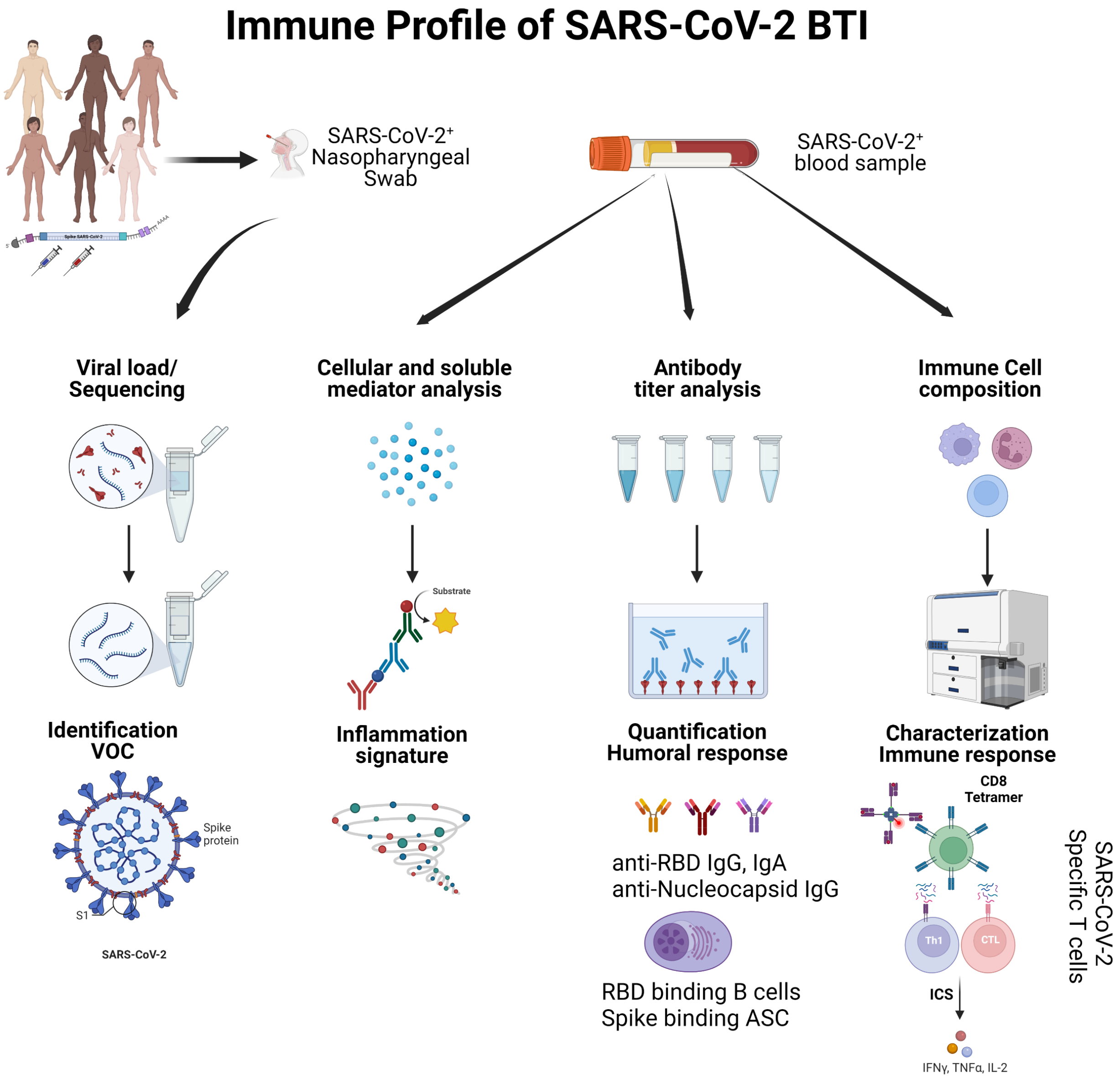

Supplementary Fig. 2. Symptoms and Inflammation during Omicron and Delta breakthrough infection

a. Self-reported symptoms of Omicron- (top) and Delta-(bottom) BTI at the time of inclusion. Duration of symptoms and PCR cycle threshold (Ct) values from the initial SARS-CoV-2 diagnostic tests are indicated. **b.** Acute phase proteins were measured in the serum (first row) and inflammatory markers were quantified in the plasma (second and third rows) of vaccinated individuals-either health care workers or healthy donors (4 months since second dose of vaccine), Omicron BTI (8-13 d after symptom debut), Delta BTI (5-24 d), and unvaccinated COVID-19 convalescent (3-6 months post-infection). Statistical analyses were performed by Mann-Whitney Test, P values are indicated. **c.** Systemic inflammatory signature of included patients. Correlogram describes the potential interaction between the different molecules measured in the serum during acute Omicron BTI (anti-Trypsin, LDH, Albumin, Transferrin, Ferritin, Haptoglobin, CRP, IFNα, IL-6, IL-18), or in the plasma (Calprotectin, CXCL4, D-Dimer, Fibrinogen, Galectin-9, GDF-15, IP-10, LBP, MPO, sCD14 and Zonulin) and cellular ratio associated with COVID-19 infection in the peripheral blood such as $CD4^{+}/CD8^{+}$, LMR (Lymphocytes/ Monocytes Ratio), and NLR (Neutrophils/Lymphocytes Ratio). The correlations are represented by a cold to hot heatmap (blue= negative correlation and red= positive correlation) and the significant combinations are indicated by an asterisk (* and ** for $p<0.05$ and $p<0.01$), see Methods for statistics. **d.** Homeostasis of immune cells during Omicron BTI**.** Cells counts were obtained by the staining of fresh blood in Trucount Tubes (BD Bioscience), Mann-Whitney Test, P value is indicated. **e.** Inflammatory signature of Omicron and Delta breakthrough infection. Principal component analyses (PCA) for individual patients according to the inflammatory profile measured in **b**. Each circle represents one individual patient position on the first two dimensions (PC1 and PC2). Delta and Omicron BTI are represented in red and green respectively and compared to vaccinated healthy donors in blue. **f.** Quantification of the Omicron BTI inflammatory signature. Volcano plot summarized the significant fold change in each marker for the Omicron BTI vs controls and the significance of these fold changes. Statistically significant changes in marker expression is represented by different colors with * and ** for $p<0.05$ and $p<0.01$ respectively. **g.** Quantification of the Delta and Omicron BTI vs convalescent COVID-19 inflammatory signature. Volcano plot summarizes the significant fold change in the serum and plasma of Delta BTI for each molecule and the significance of these fold changes in comparison with healthy donors (left) or Omicron BTI (middle). COVID-19 convalescent patients were compared to vaccinated healthy donors (right). Source data are provided as a Source Data file.

Supplementary Figure 2

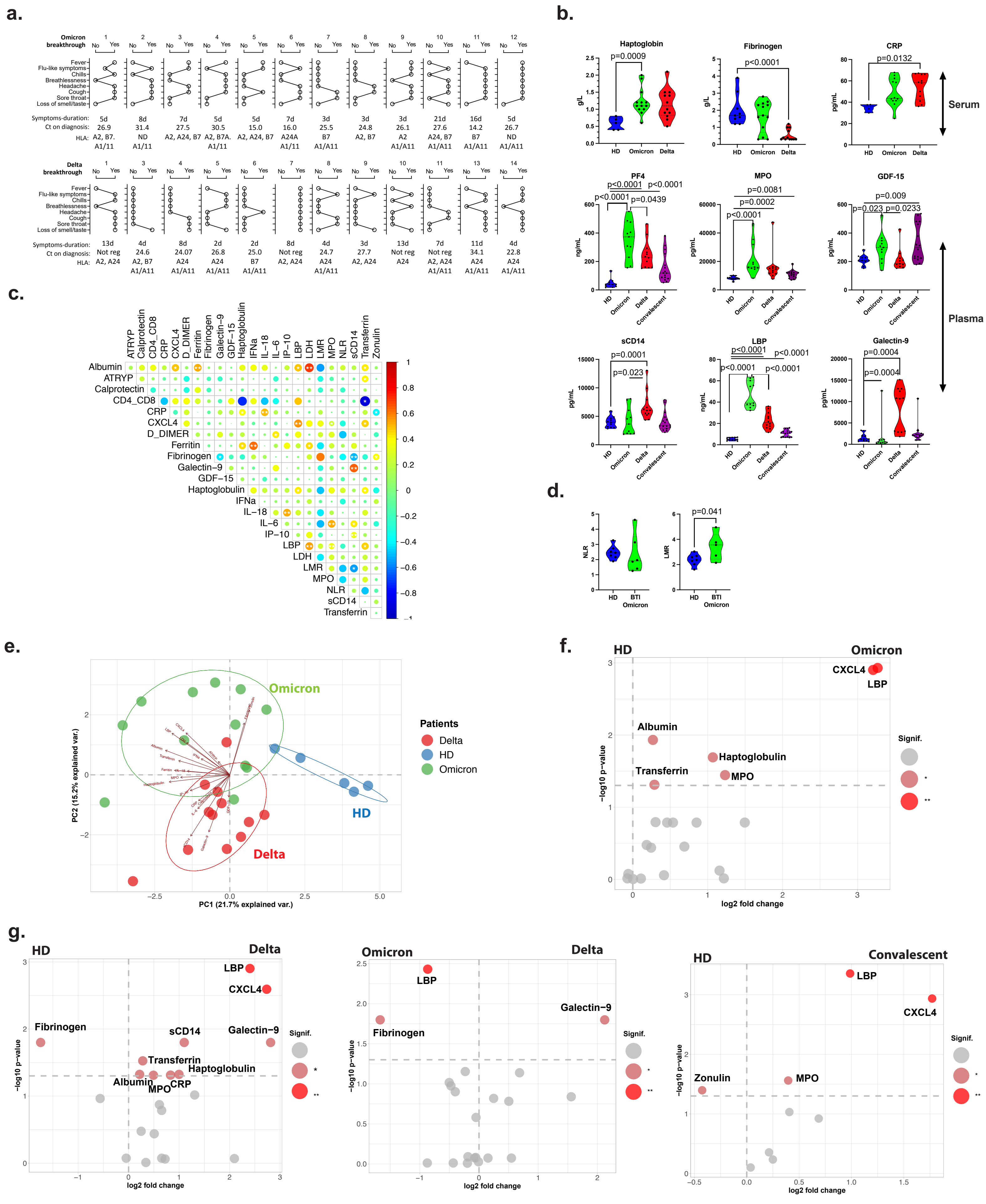

Supplementary Figure 3. $CD8^+$ T cell immunity during Omicron and Delta BTI

a. Signature of Spike-specific $CD8^+$ T cells during Omicron BTI. The expression of surface markers by antigen-specific CD8 T cells were compared between Spike-specific $dextramer^+$ cells detected in healthy donors (blue) and in Omicron- BTI (green). Mann-Whitney test P values are shown. **b.** Signature of Spike-specific $CD8^+$ T cells during BTI vs vaccinated healthy donor controls. High dimensional analysis of individual patients according to the phenotype of Spike-specific Dextramers in principal component analysis (PCA) biplots. Each dot represents one individual patient analysis. Delta- and Omicron-infected patients are represented in red and green respectively and compared to vaccinated healthy donors (blue). **c.** Analysis of log P value vs. fold change, volcano plots of Spike-specific CD8 T cells during Delta BTI vs HD (left), and Delta vs. Omicron BTI (right). Significantly upregulated markers are indicated in darker shades of red. **d.** Distribution of SARS-CoV-2 specific T cells and control-virus specific $CD8^+$ T cells in tSNE plot. The phenotype of Spike-, Nucleocapsid-, and ORF-specific $CD8^+$ T cells was compared to immune profile of CMV- and EBV-specific $CD8^+$ T cells from our entire cohort. The profile of each virus-specific $CD8^+$ T cell was visualized by tSNE representation with CMV-, EBV-, SARS-CoV-2 non-spike-ORF/N), and Spike-specific CTLs in red, blue, green and purple respectively. **e.** Immune phenotype ex vivo of non-spike (ORF/N) SARS-CoV-2 specific $CD8^+$ T cells. A cold to hot heatmap represents the scaled frequency of each individual marker expressed by non-Spike antigen-specific $CD8^+$ T cells. Patient. HLA type and time post-infection are indicated in top two rows-Delta BTI, Omicron BTI, vaccinated healthy donors (HD); (HLA subtypes). The identification of marker types as subsets (Activation, Effector, Exhaustion, Memory) are indicated in the leftmost column. **f.** PCA Biplot analysis of non-Spike SARS-CoV-2 specific $CD8^+$ T cells during Delta and Omicron BTI vs vaccinated HD. **g.** Analysis of log p-value vs fold change, volcano plots of non-Spike SARS-CoV-2 specific $CD8^+$ T cells during Omicron BTI vs vaccinated HD (left), Delta BTI vs vaccinated HD (middle) and Delta BTI vs Omicron BTI (right). Significant markers are indicated in darker shades of red. Source data are provided as a Source Data file.

Supplementary Figure 3

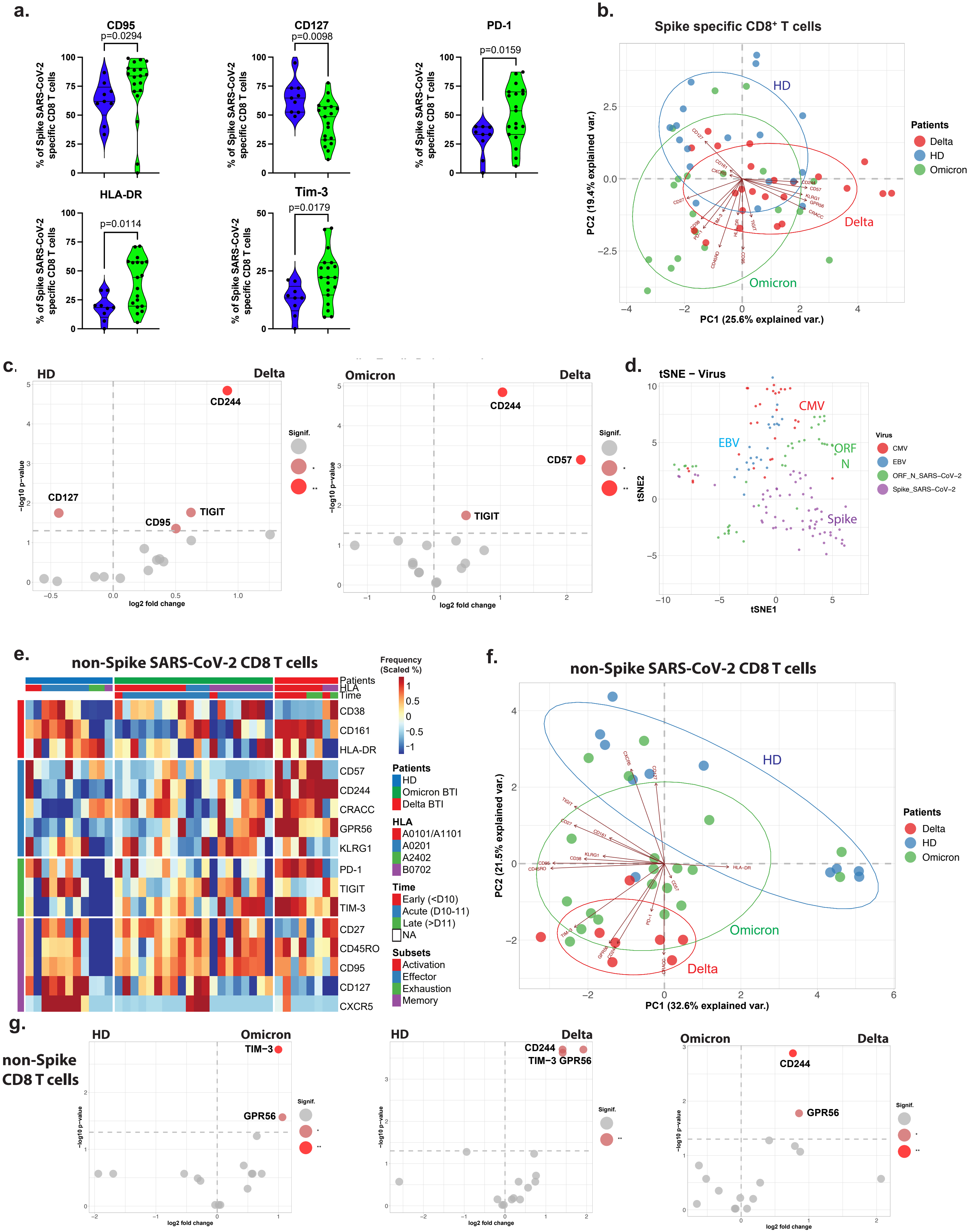

Supplementary Figure 4. CD8$^+$ T cells analysis of granzyme B and perforin
a. Immune phenotype ex vivo of Spike and **b**. non-spike (ORF/N) SARS-CoV-2 specific CD8$^+$ T cells. A cold to hot heatmap represents the scaled frequency of each individual marker expressed by a. Spike and b. non-Spike antigen-specific CD8$^+$ T cells. The distribution of markers and patients were automatically performed by unsupervised hierarchical clustering. Patient and HLA type are indicated in top two rows-Delta BTI, Omicron BTI, vaccinated healthy donors (HD); (HLA subtypes). The identification of marker types as subsets (Activation, Effector, Exhaustion, Memory) are indicated in the leftmost column. **c.** Functionality of SARS-CoV-2 specific CD8$^+$ T cells. Activation (CD137) and expression of granzyme B (CD137$^+$GZM-B$^+$, left panel) and perforin (CD137$^+$Perforin$^+$, right panel) in CD8$^+$ T cells responding to the stimulation in vitro by Spike (WT) peptides, left; middle: pooled overlapping SARS-CoV-2 membrane (M) and nucleoprotein (N) peptides, and ORF peptides-See Methods Table 2) –and right: the whole proteome of WT SARS-CoV-2 (S, M, N, E, O), see Methods. Mann Whitney Test P values are shown. Source data are provided as a Source Data file.

Supplementary Figure 4

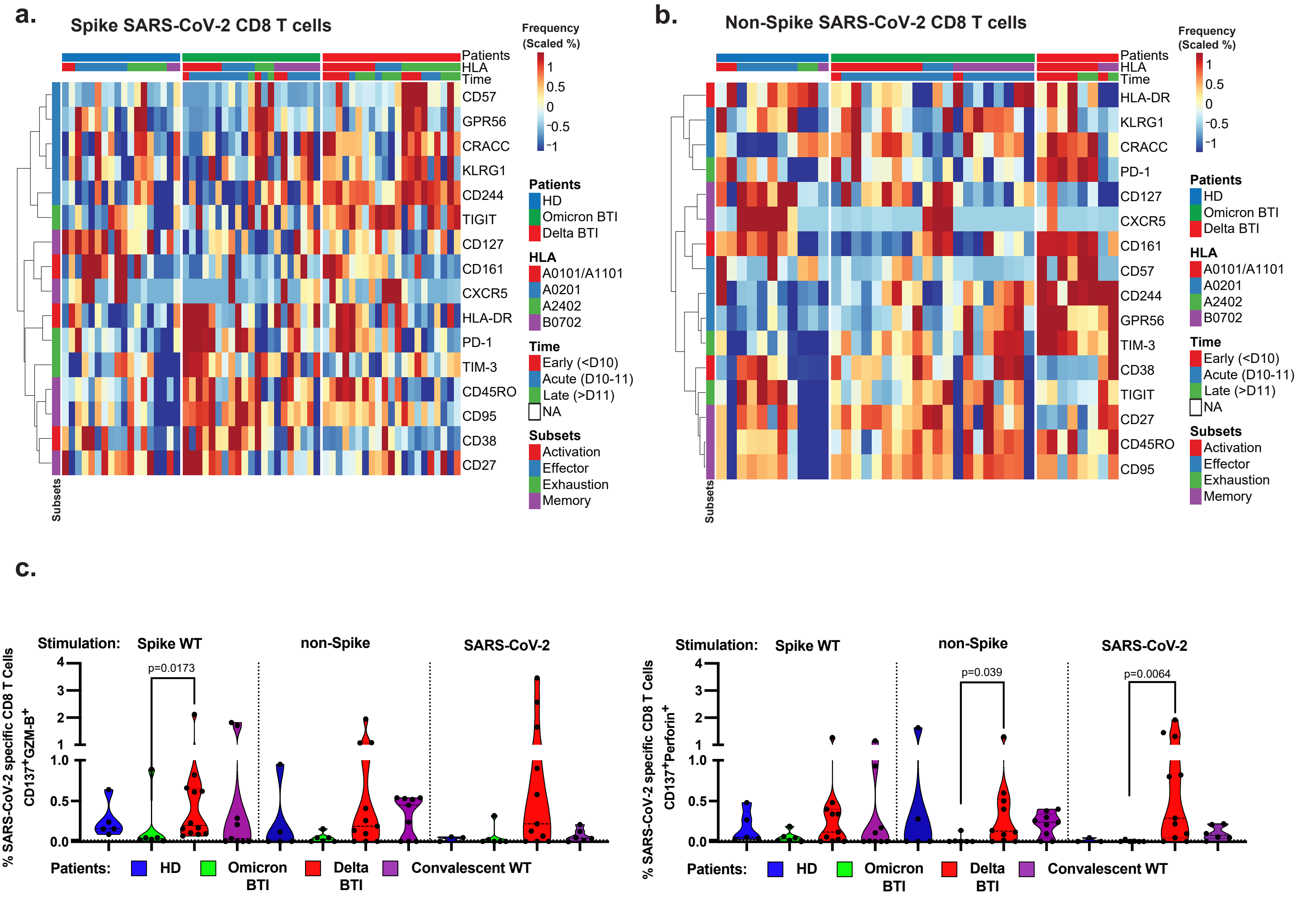

Supplementary Figure 5. Non-classical T cells during Omicron and Delta BTI
Quantification of innate T cells and CD4/8 T cell ratio by mass cytometry. Left panel: γδ T cells were identified from cryo-preserved PBMCs with the expression of the Pan-γδ TCR. Middle panel - MAIT cells were defined by the co-expression of CD161 and TCR Vα7.2 during Delta and Omicron BTI vs vaccinated HD. Right panel: Ratio of CD4/CD8 CD3+ T cells. Mann Whitney Test P values are shown. See also gating strategy in extended Figure 8.

Supplementary Figure 5

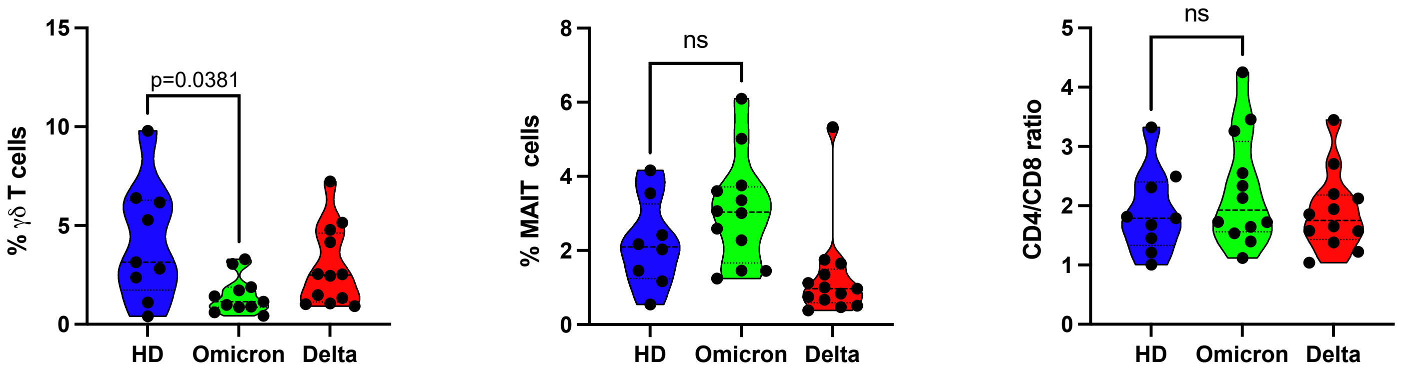

Supplementary Figure 6. CD4$^+$ T helper cell immunity during Omicron and Delta BTI

a. Phenotype of Follicular helper CD4$^+$ T cells during BTI. Mass cytometry staining of T_{FH} cells-identified as CXCR5$^+$ CD4$^+$ T cells in vaccinated infected patients. Cells from representative patients of each group (n=6) were concatenated and visualized by tSNE generated by analysis of the following markers: CCR4, CCR6, CCR7, CD25, CD27, CD38, CD45RA, CD56, CD57, CD71, CD85j, CD95, CD127, CD160, CD161, PD-1, CRTH2, CXCR3, HLA-DR, TIGIT, TIM-3. The intensity of each marker is represented by a heatmap. The Phenograph algorithm was used to automatically identify the different clusters (see phenograph plot in lower row). Distribution of cells from patient categories are shown-right in lower row). Distribution of T_{FH} cells in tSNE plot is shown for markers as indicated. **b.** Distribution of cells within clusters identified by Phenograph in a. for Omicron and Delta BTI and vaccinated HD, **c.** Overview and spatial visualization of T_{FH} cells from vaccinated HD, Omicron and Delta BTI. Localization is based on specific co-expression of markers as shown in a. **d.** Immune phenotype of T_{FH} cells. A cold to hot heatmap represents the scaled intensity of each individual metal-tagged marker expressed by clusters of T_{FH} cells identified in a. The distribution of markers and clusters were automatically performed by unsupervised hierarchical clustering. **e.** Identification of SARS-CoV-2 specific CD4 T cells by flow cytometry. Representative dot plots of CD4 T cells left unstimulated, or after stimulation with overlapping peptides from Spike; after stimulation with non-spike peptides-pooled overlapping SARS-CoV-2 membrane (M) and nucleoprotein (N) peptides, and ORF peptides; or stimulated with full SARS-CoV-2-the whole proteome of WT SARS-CoV-2 (S, M, N, E, O). Cytostim was used as positive control. CD154 vs CD137, TNF, IFN-γ, IL-2 are shown. **f**. Quantification of SARS-CoV-2 specific CD4 T cells by flow cytometry. Cells were stimulated by Spike peptide as in e. or left unstimulated (US). Specific responders were quantified as CD154$^+$CD137$^+$, CD154$^+$IFN-γ^+ or CD154$^+$TNF$^+$. **g**. SARS-CoV-2 specific responses of CD4$^+$ Th cells in response to peptides as indicated. Left: Spike(S) peptides. Middle: non-spike peptides (M, N, O). Right: the whole proteome of WT SARS-CoV-2 (S, M, N, E, O). CD154$^+$IL-2$^+$ cells are shown. The statistical analysis was performed with Wilcoxon test on paired samples for the comparison between unstimulated and peptides stimulated samples and with rank Mann-Whitney for the comparison between different groups. The P values are shown. Source data are provided as a Source Data file.

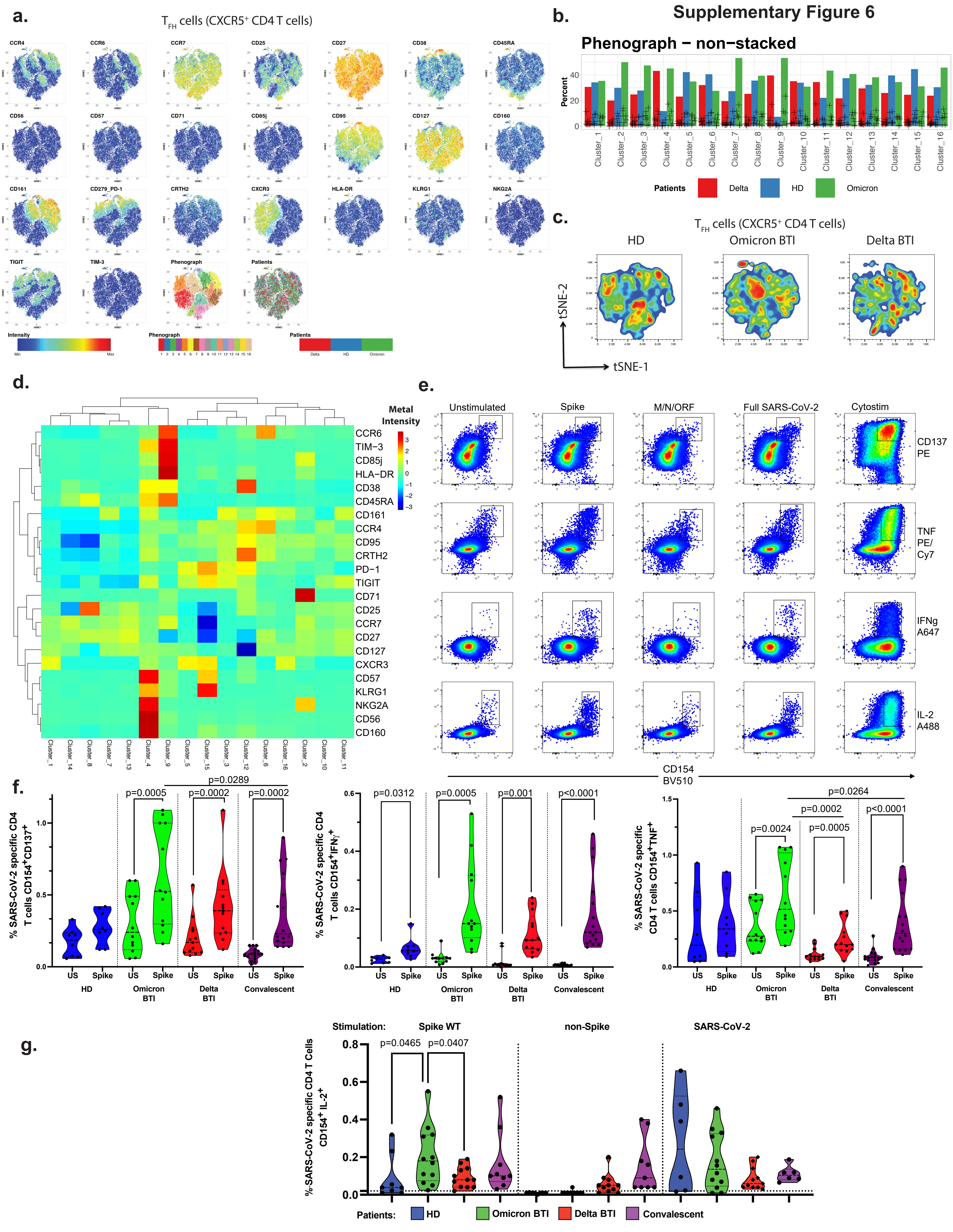
Supplementary Figure 6
a.
TFH cells (CXCR5+ CD4 T cells)
CCR4
CCR6
CCR7
CD25
CD27
CD38
CD45RA
CD56
CD57
CD71
CD85j
CD95
CD127
CD160
CD161
CD279_PD-1
CRTH2
CXCR3
HLA-DR
KLRG1
NKG2A
TIGIT
TIM-3
Phenograph
Patients
Intensity
Min
Max
Delta
HD
Omicron
b.
Phenograph − non−stacked
Percent
Patients
Delta
HD
Omicron
c.
TFH cells (CXCR5+ CD4 T cells)
HD
Omicron BTI
Delta BTI
tSNE-2
tSNE-1
d.
Metal Intensity
CCR6
TIM−3
CD85j
HLA−DR
CD38
CD45RA
CD161
CCR4
CD95
CRTH2
PD−1
TIGIT
CD71
CD25
CCR7
CD27
CD127
CXCR3
CD57
KLRG1
NKG2A
CD56
CD160
e.
Unstimulated
Spike
M/N/ORF
Full SARS-CoV-2
Cytostim
CD137 PE
TNF PE/Cy7
IFNg A647
IL-2 A488
CD154 BV510
f.
% SARS-CoV-2 specific CD4 T cells CD154+CD137+
p=0.0005
p=0.0289
p=0.0002
p=0.0002
US
Spike
HD
Omicron BTI
Delta BTI
Convalescent
% SARS-CoV-2 specific CD4 T cells CD154+IFNγ+
p=0.0312
p=0.0005
p=0.001
p<0.0001
% SARS-CoV-2 specific CD4 T cells CD154+TNF+
p=0.0264
p=0.0002
p<0.0001
p=0.0024
p=0.0005
g.
Stimulation:
Spike WT
non-Spike
SARS-CoV-2
p=0.0465
p=0.0407
% SARS-CoV-2 specific CD4 T Cells CD154+ IL-2+
Patients:
HD
Omicron BTI
Delta BTI
Convalescent

Supplementary Figure 7: B cell and humoral immunity during Omicron and Delta BTI

a. Phenotype of Spike-binding B cells during breakthrough infection. Dot plots of live singlet total B cells from one representative healthy donor (top two rows) and one Omicron-infected individual (bottom two rows) are shown. Dark blue dots are Spike-binding B cells. Grey outlined plots show non-binding B cells. Surface molecules (IgG, IgD, CD21, CD24, CD27, CD38, CD71) and intra-cellular transcription factors (IRF-4 and Blimp-1) are represented on the X axis and Spike-binding on the Y axis. **b.** Distribution of class switched IgG vs IgM/D of Spike-binding B cells in pie charts as indicated. Spike binding B cells that are IgG vs Non-IgG are shown in violin plots. HD (blue), Omicron BTI (green) and Delta BTI (red) are shown for RBD-Spike binders (left) and non-RBD Spike binders (right). Mann Whitney test P values are shown. **c.** Humoral response and clinical parameters. The correlations between anti-RBD IgG (BAU/mL) and the frequency of Spike-binding B cells, anti-RBD IgA (BAU/mL), anti-Nucleocapsid IgG (BAU/mL), the age of patients, and the inclusion time post-infection were calculated by a Spearman's rank order test, r values and P values are indicated on the graphs. Source data are provided as a Source Data file.

Supplementary Figure 7

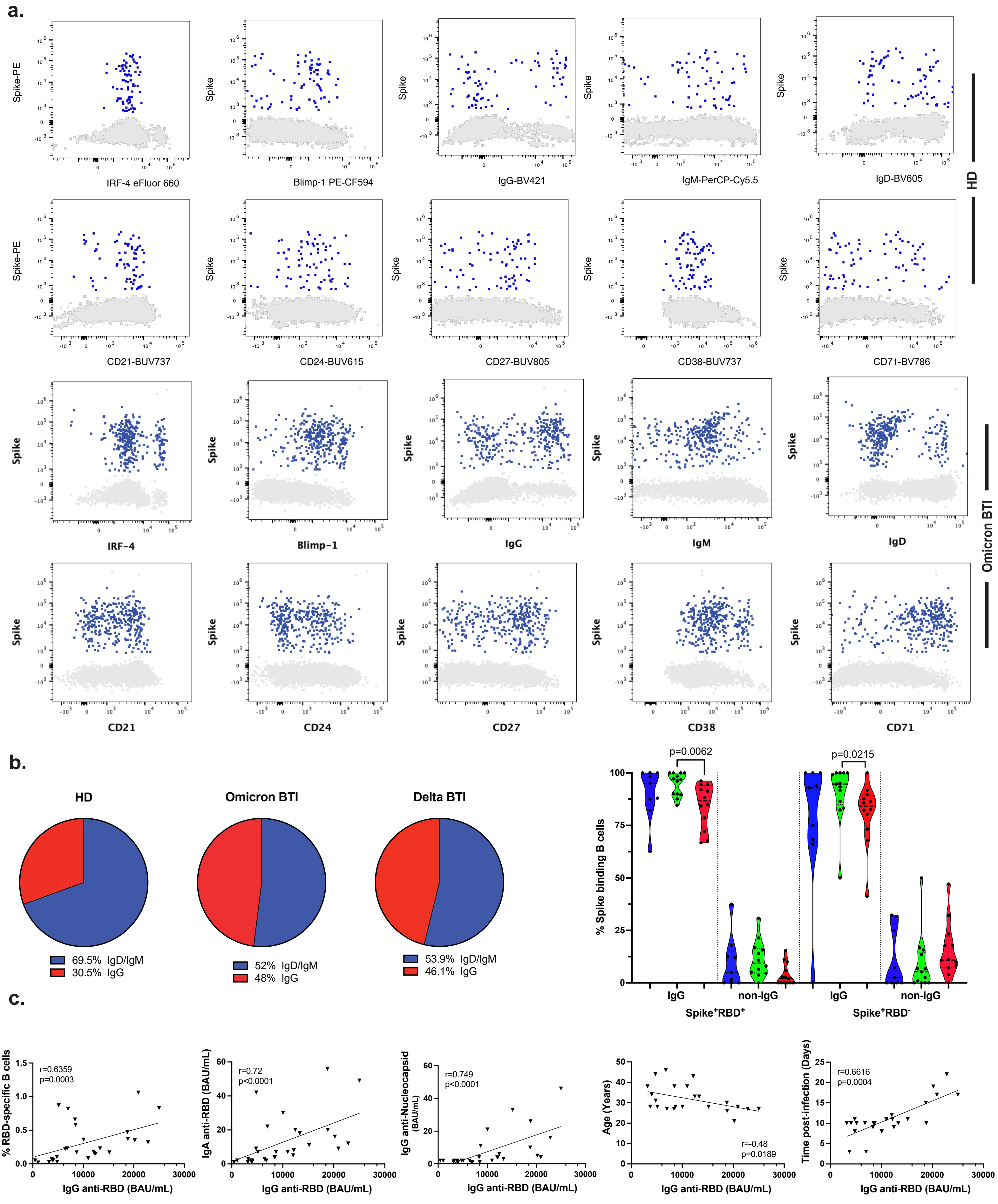

Supplementary Figure 8: Gating strategy

a. Identification of Virus-specific CD8 T cells by flow cytometry. Illustrative example of Class I restricted multimers targeting Spike-, Nucleocapsid- and EBV- specific CD8 T cells is displayed. **b**. Identification of Follicular CD4 T cells by mass cytometry. Individual donor cells were de-barcoded according to their unique combination of double CD45 expression. T_{FH} cells were identified as $CXCR5^+$ cells in total CD4 T cells after exclusion of dead, non-T cells, γδ T cells and MAIT cells ($CD161^+$TCR $V\alpha7.2^+$). **c**. Identification of Spike-binding B cells by flow cytometry. Spike and Spike RBD SARS-CoV-2 binding B cells were identified in total B cells after exclusion of doublets, dead, and $Lineage^+$ (Monocytes, NK cells and T cells). The phenotype of B cells was also studied by high parameters flow cytometry to detail naïve (IgD^+) and Switched (IgG^+) memory B cells.

Supplementary Figure 8

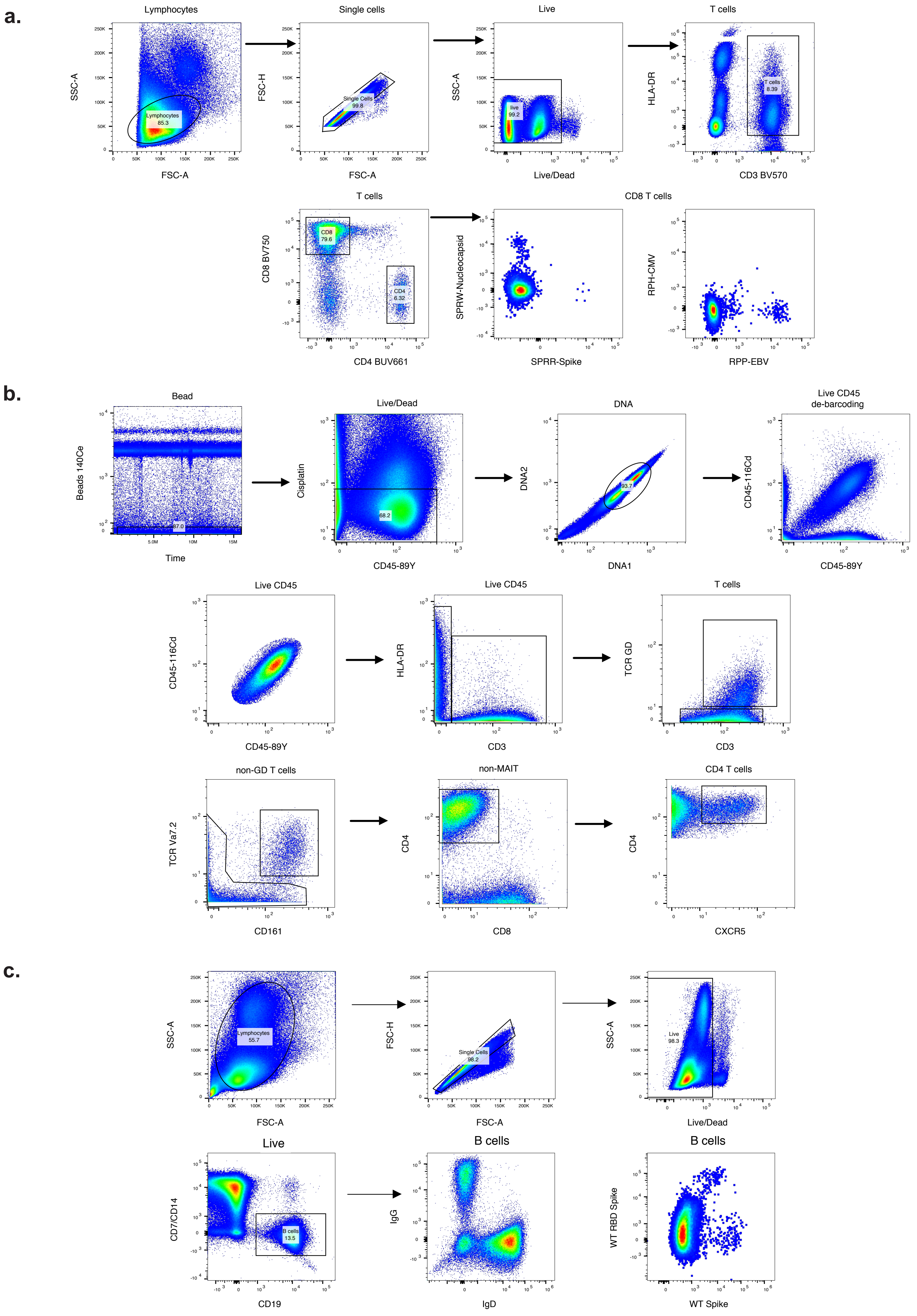

Supplementary Figure 9: Examples of peptide:HLA multimer stains of CD8$^+$ T cells
Cells were stained as described in the Methods section with peptide:HLA multimers (either dextramers from Immudex or Flex T tetramers from BioLegend), see Table 2 for peptide:HLA combinations. Dotplots show gated CD8$^+$ T cells (see Supplementary Figure 8 for gating), HLA peptide multimers as indicated vs. PD-1 are shown for Omicron or Delta BTI, or HD.

Supplementary Figure 9

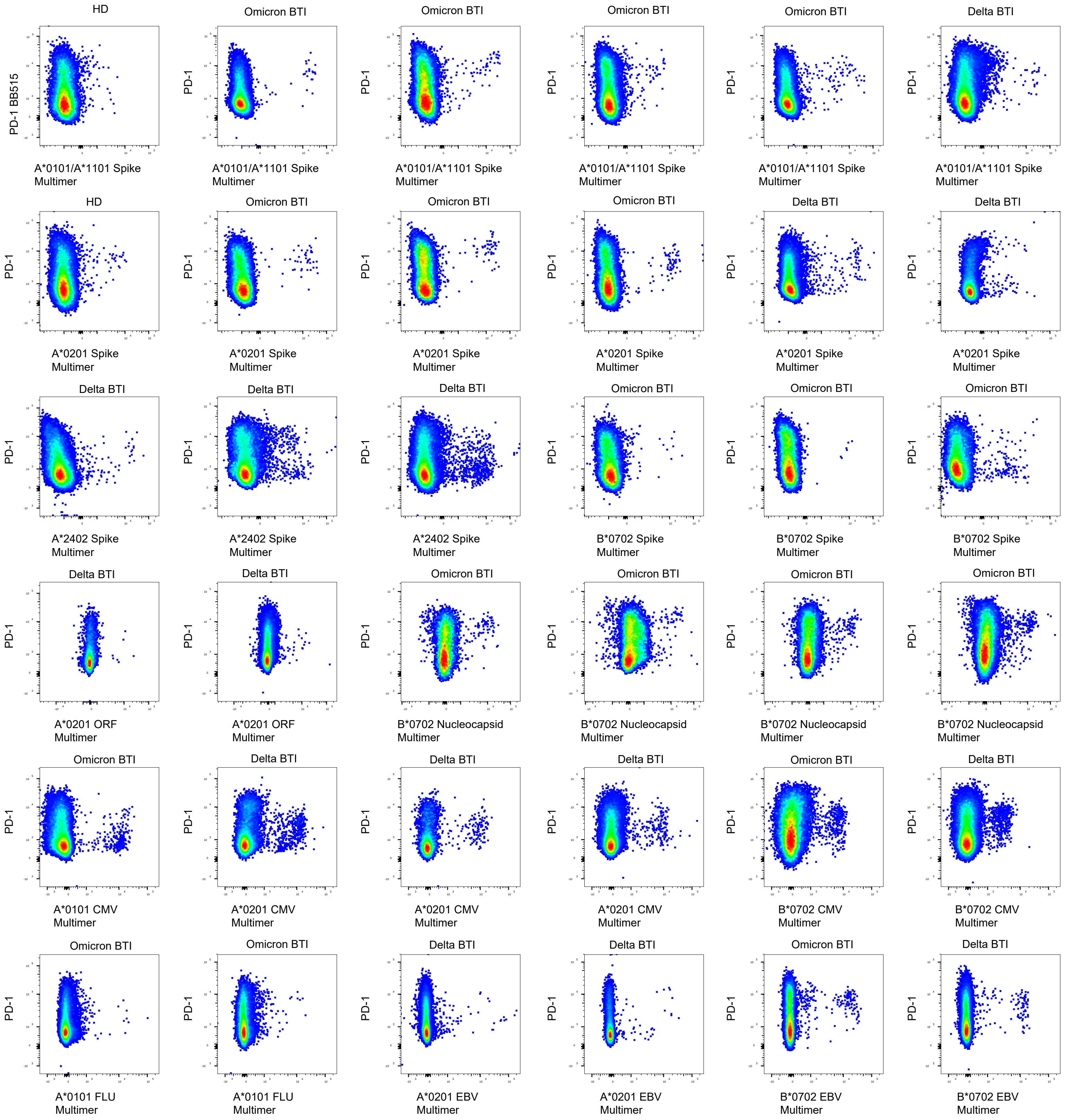

Extended Methods and Reference

The following immunodominant peptide/HLA have been described as referenced: HLA-A*0101-LTDEMIAQY[1-6], HLA-A*0201 -YLQPRTFLL[2,4,7-10], HLA-A*2402-QYIKWPWYI[2-4,6,7], HLA-B*0702 - SPRRARSVA[4,8,11], HLA-A*0101-YTNSFTRGVY[1,4], HLA-A*0201-LITGRLQSL[10-13] and RLNEVAKNL[5,10,11,14], HLA-A*2402-NYNYLYRLF[1,2,6,15], and HLA-B*0702-APHGVVFL[2,5]. Non-Spike derived peptides were HLA-A*0101-ORF3a, FTSDYYQLY[2,4,11,16] and ORF1ab, TTDPSFLGRY[3-5,7], HLA-A*0201-ORF3a, LLYDANYFL[2,7,8,11], HLA-A*2402-ORF3a, VYFLQSINF[2,3,7], and HLA-B*0702-Nucleoprotein, SPRWYFYYL[7,8]. CMV derived epitopes were for HLA-A*0101-DNA polymerase processivity factor, VTEHDTLLY[2,6,17], HLA-A*0201-65 kDa phosphoprotein, NLVPMVATV[2,18], HLA-A*2402-65 kDa phosphoprotein, QYDPVAALF[2,19], and HLA-B*0702-65 kDa phosphoprotein, RPHERNGFTVL[2,20]. EBV derived epitopes were for HLA-A*0201-EBV LMP2, FLYALALLL and HLA-B*0702-EBV antigen 3, RPPIFIRRL[2,21]. A Flu peptide was for HLA-A*0101-Nucleoprotein, Influenza A virus CTELKLSDY[22].

1 Tarke, A. *et al.* Comprehensive analysis of T cell immunodominance and immunoprevalence of SARS-CoV-2 epitopes in COVID-19 cases. *Cell Rep Med* **2**, 100204, doi:10.1016/j.xcrm.2021.100204-2021).

2 Kared, H. *et al.* SARS-CoV-2-specific CD8+ T cell responses in convalescent COVID-19 individuals. *J Clin Invest* **131**, doi:10.1172/jci145476-2021).

3 Nelde, A. *et al.* SARS-CoV-2-derived peptides define heterologous and COVID-19-induced T cell recognition. *Nat Immunol* **22**, 74-85, doi:10.1038/s41590-020-00808-x-2021).

4 Saini, S. K. *et al.* SARS-CoV-2 genome-wide T cell epitope mapping reveals immunodominance and substantial CD8(+) T cell activation in COVID-19 patients. *Sci Immunol* **6**, doi:10.1126/sciimmunol.abf7550-2021).

5 Gangaev, A. *et al.* Abstract S05-01: Profound CD8 T-cell responses towards SARS-CoV-2 OFR1ab in COVID-19 patients. *Clinical Cancer Research* **26**, S05-01-S05-01, doi:10.1158/1557-3265.Covid-19-s05-01-2020).

6 Gangaev, A. *et al.* Identification and characterization of a SARS-CoV-2 specific CD8(+) T cell response with immunodominant features. *Nat Commun* **12**, 2593, doi:10.1038/s41467-021-22811-y-2021).

7 Ferretti, A. P. *et al.* Unbiased Screens Show CD8⁺ T Cells of COVID-19 Patients Recognize Shared Epitopes in SARS-CoV-2 that Largely Reside outside the Spike Protein. *Immunity* **53**, 1095-1107.e1093, doi:10.1016/j.immuni.2020.10.006-2020).

8 Sekine, T. *et al.* Robust T Cell Immunity in Convalescent Individuals with Asymptomatic or Mild COVID-19. *Cell* **183**, 158-168.e114, doi:10.1016/j.cell.2020.08.017-2020).

9 Shomuradova, A. S. *et al.* SARS-CoV-2 Epitopes Are Recognized by a Public and Diverse Repertoire of Human T Cell Receptors. *Immunity* **53**, 1245-1257.e1245, doi:10.1016/j.immuni.2020.11.004-2020).

10 Rha, M. S. *et al.* PD-1-Expressing SARS-CoV-2-Specific CD8(+) T Cells Are Not Exhausted, but Functional in Patients with COVID-19. *Immunity* **54**, 44-52.e43, doi:10.1016/j.immuni.2020.12.002-2021).

11 Schulien, I. *et al.* Characterization of pre-existing and induced SARS-CoV-2-specific CD8(+) T cells. *Nat Med* **27**, 78-85, doi:10.1038/s41591-020-01143-2-2021).

12 Rha, M.-S. *et al.* PD-1-Expressing SARS-CoV-2-Specific CD8⁺ T Cells Are Not Exhausted, but Functional in Patients with COVID-19. *Immunity* **54**, 44-52.e43, doi:10.1016/j.immuni.2020.12.002-2021).

13 Chour, W. *et al.* Shared Antigen-specific CD8⁺ T cell Responses Against the SARS-COV-2 Spike Protein in HLA-A*02:01 COVID-19 Participants. *medRxiv*, 2020.2005.2004.20085779, doi:10.1101/2020.05.04.20085779-2020).

14 Nielsen, S. S. *et al.* SARS-CoV-2 elicits robust adaptive immune responses regardless of disease severity. *EBioMedicine* **68**, 103410, doi:10.1016/j.ebiom.2021.103410-2021).

15 Sahin, U. *et al.* BNT162b2 vaccine induces neutralizing antibodies and poly-specific T cells in humans. *Nature* **595**, 572-577, doi:10.1038/s41586-021-03653-6-2021).

16 Peng, Y. *et al.* Broad and strong memory CD4(+) and CD8(+) T cells induced by SARS-CoV-2 in UK convalescent individuals following COVID-19. *Nat Immunol* **21**, 1336-1345, doi:10.1038/s41590-020-0782-6-2020).

17 Tey, S. K., Goodrum, F. & Khanna, R. CD8+ T-cell recognition of human cytomegalovirus latency-associated determinant pUL138. *J Gen Virol* **91**, 2040-2048, doi:10.1099/vir.0.020982-0-2010).

18 Diamond, D. J., York, J., Sun, J. Y., Wright, C. L. & Forman, S. J. Development of a candidate HLA A*0201 restricted peptide-based vaccine against human cytomegalovirus infection. *Blood* **90**, 1751-1767-1997).

19 Kuzushima, K., Hayashi, N., Kimura, H. & Tsurumi, T. Efficient identification of HLA-A*2402-restricted cytomegalovirus-specific CD8(+) T-cell epitopes by a computer algorithm and an enzyme-linked immunospot assay. *Blood* **98**, 1872-1881, doi:10.1182/blood.v98.6.1872-2001).

20 Hebart, H. *et al.* A CTL epitope from human cytomegalovirus IE1 defined by combining prediction of HLA binding and proteasomal processing is the target of dominant immune responses in patients after allogeneic stem cell transplantation. *Exp Hematol* **31**, 966-973, doi:10.1016/s0301-472x(03)00203-0-2003).

21 Weingarten-Gabbay, S. *et al.* Profiling SARS-CoV-2 HLA-I peptidome reveals T cell epitopes from out-of-frame ORFs. *Cell* **184**, 3962-3980.e3917, doi:10.1016/j.cell.2021.05.046-2021).

22 DiBrino, M. *et al.* HLA-A1 and HLA-A3 T cell epitopes derived from influenza virus proteins predicted from peptide binding motifs. *J Immunol* **151**, 5930-5935-1993).
