# Supplementary material for: Immune responses in Omicron SARS-CoV-2 breakthrough infection in vaccinated adults
Source: Nat Commun. 2022 Jul 18;13:4165. doi: 10.1038/s41467-022-31888-y (PMC9293966; doi:10.1038/s41467-022-31888-y)
Supplement: Supplementary file 2 — Reporting Summary [file 41467_2022_31888_MOESM2_ESM.pdf]

nature portfolio

Corresponding author(s): Hassen Kared, Ludvig Munthe

Last updated by author(s): 16th June 2022

Reporting Summary

Nature Portfolio wishes to improve the reproducibility of the work that we publish. This form provides structure for consistency and transparency in reporting. For further information on Nature Portfolio policies, see our Editorial Policies and the Editorial Policy Checklist.

Statistics

For all statistical analyses, confirm that the following items are present in the figure legend, table legend, main text, or Methods section.

n/a	Confirmed	
☐	☒	The exact sample size (*n*) for each experimental group/condition, given as a discrete number and unit of measurement
☐	☒	A statement on whether measurements were taken from distinct samples or whether the same sample was measured repeatedly
☐	☒	The statistical test(s) used AND whether they are one- or two-sided<br>*Only common tests should be described solely by name; describe more complex techniques in the Methods section.*
☐	☒	A description of all covariates tested
☐	☒	A description of any assumptions or corrections, such as tests of normality and adjustment for multiple comparisons
☐	☒	A full description of the statistical parameters including central tendency (e.g. means) or other basic estimates (e.g. regression coefficient) AND variation (e.g. standard deviation) or associated estimates of uncertainty (e.g. confidence intervals)
☐	☒	For null hypothesis testing, the test statistic (e.g. F, t, r) with confidence intervals, effect sizes, degrees of freedom and P value noted<br>*Give P values as exact values whenever suitable.*
☒	☐	For Bayesian analysis, information on the choice of priors and Markov chain Monte Carlo settings
☒	☐	For hierarchical and complex designs, identification of the appropriate level for tests and full reporting of outcomes
☒	☐	Estimates of effect sizes (e.g. Cohen's d, Pearson's r), indicating how they were calculated

Our web collection on statistics for biologists contains articles on many of the points above.

Software and code

Policy information about availability of computer code

Data collection	Attune™ NxT Software for the Attune™ NxT Flow Cytometer. Facs Diva, BD FACSymphony™.
Data analysis	CYTOGRAPHER® (ImmunoScape cloud based analytical software), GraphPad Prism version 9 (GraphPad Software) and FlowJo v10 software (BD Life Sciences).

For manuscripts utilizing custom algorithms or software that are central to the research but not yet described in published literature, software must be made available to editors and reviewers. We strongly encourage code deposition in a community repository (e.g. GitHub). See the Nature Portfolio guidelines for submitting code & software for further information.

Data

Policy information about availability of data

All manuscripts must include a data availability statement. This statement should provide the following information, where applicable:

- Accession codes, unique identifiers, or web links for publicly available datasets
- A description of any restrictions on data availability
- For clinical datasets or third party data, please ensure that the statement adheres to our policy

The datasets generated and analysed during the current study are available from the corresponding author on reasonable request. The patient samples are not available on request due to restricting ethical and legal approvals. Source data are available in a Source Data File.

Field-specific reporting

Please select the one below that is the best fit for your research. If you are not sure, read the appropriate sections before making your selection.

☒ Life sciences ☐ Behavioural & social sciences ☐ Ecological, evolutionary & environmental sciences

For a reference copy of the document with all sections, see nature.com/documents/nr-reporting-summary-flat.pdf

Life sciences study design

All studies must disclose on these points even when the disclosure is negative.

Sample size

We have included 13-14 cases in two groups (Omicron breakthrough Covid-19 vs Delta breakthrough Covid-19) and in fully vaccinated controls (healthy donors, health care workers). The design is a case vs case vs control in a high dimensionality observational analysis. We did not perform sample size calculations but chose n>10 for functional analyses as this is most often sufficient to discover differences in groups that are relevant for the current type of study.

Data exclusions

One of the Delta cases was found to be an Omicron infected individual and was therefore recategorized.

Replication

The data were replicated with further detailed extended analysis, e.g. further functional analysis of additional readouts, additional peptides that were utilized in flow cytometry assays (peptide-HLA-multimers) for further validation.

Randomization

There was no intervention and no randomization in the design

Blinding

Acquisition of samples was blinded in assays measuring inflammatory markers as technicians had no knowledge of the sample key. The analysis of results was not blinded in the design analysis as HLA-type (various alleles, up to three) was required to assay samples.

Reporting for specific materials, systems and methods

We require information from authors about some types of materials, experimental systems and methods used in many studies. Here, indicate whether each material, system or method listed is relevant to your study. If you are not sure if a list item applies to your research, read the appropriate section before selecting a response.

Materials & experimental systems

n/a	Involved in the study	
☐	☒	Antibodies
☒	☐	Eukaryotic cell lines
☒	☐	Palaeontology and archaeology
☒	☐	Animals and other organisms
☐	☒	Human research participants
☒	☐	Clinical data
☒	☐	Dual use research of concern

Methods

n/a	Involved in the study	
☒	☐	ChIP-seq
☐	☒	Flow cytometry
☒	☐	MRI-based neuroimaging

Antibodies

Antibodies used

BB515 Mouse Anti-Human CD279 (PD-1) Clone EH12.1, 50 tests, Catalog # 565936, BD Biosciences. PerCP-eFluor 710, KLRG1 Monoclonal Antibody (13F12F2), 5 µL (0.25 µg)/test, Catalog # 46-9488-42, eBioscience. PE/Cyanine7 anti-human GPR56, Clone CG4, 100 tests, Catalog # 358206, BioLegend. Alexa Fluor 700 anti-human CD244 (2B4), clone C1.7, 100 tests, Catalog # 329526, BioLegend. APC/Cyanine7 anti-human HLA-DR, clone L243, 100 tests, Catalog # 307618 BioLegend. BV480 Rat Anti-Human CXCR5 (CD185) (Clone: RF8B2), 100 tests, Catalog # 566142 BD Biosciences. BB515 Mouse Anti-Human CD38, clone HIT2, 100 tests, Catalog # 564498 BD Biosciences. BV570 anti-human CD3, BioLegend, 100 tests, Catalog # 300436. BV605 Mouse anti Human CD127, Clone HIL 7R M21, 50 tests, Catalog 562662, BD Biosciences. BV650 Mouse anti Human CD161, clone: DX12, 100 tests, Catalog # 563864, BD Biosciences. BV711 Mouse Anti-Human TIM-3 (CD366), clone 7D3, 100 tests, Catalog # 565567,BD Biosciences. BV750 Mouse Anti-Human CD8, clone SK1, 100 tests, Catalog # 747098, BD Biosciences. BV785™ anti-human CD57 Recombinant Ab, clone QA17A04, 100 tests, Catalog # 393330, BioLegend. BV421 Mouse Anti-Human CD319 (CRACC), 50 tests, Catalog # 750819, BD Biosciences. BUV395 Mouse Anti-Human TIGIT, clone 741182, 100 tests, Catalog # 741182, BD Biosciences. Live/dead™ Fixable Blue Dead Cell Stain Kit, for UV excitation, Thermo Fisher Scientific, 400 tests, Catalog # L34962. BUV563 Mouse Anti-Human CD45RO, clone UCHL1, 100 tests, Catalog # 748369, BD Biosciences. BUV615 Mouse Anti-Human CD95, clone DX, BD 100 tests, Catalog # 752346, Biosciences. BUV661 Mouse Anti-Human CD4, clone SK3, 100 tests, Catalog # 612962, BD Biosciences. BUV737 Mouse Anti-Human CD38, clone HB7, 100 tests, Catalog # 612824, BD Biosciences. BUV805 Mouse Anti-Human CD27, clone L128, 100 tests, Catalog # 751682, BD Biosciences. BUV805-Mouse Anti-Human CD14, clone M5E2, 100 tests, Catalog # 612902, BD Biosciences. BV711-Mouse Anti-Human CD19, clone HIB19, 100 tests, Catalog # 563036, BD Biosciences. BUV395-Mouse Anti-Human CD20, clone 2H7, 100 tests, Catalog # 563782, BD Biosciences. BUV737-Mouse Anti-Human CD21, clone B-ly4, 100 tests, Catalog # 612788, BD

Biosciences. BUV615-Mouse Anti-Human CD24, clone ML5, 100 tests, Catalog # 751122, BD Biosciences. BV786-Mouse Anti-Human CD27, clone L128, 100 tests, Catalog # 563327, BD Biosciences. BB515-Mouse Anti-Human CD38, clone HIT2, 100 tests, Catalog # 564498, BD Biosciences. PE-Cy-7-Mouse Anti-Human CD71, clone CY1G4, 100 tests, Catalog # 564019, BD Biosciences. BV605-Mouse Anti-Human IgD, clone IA6-2, 50 tests, Catalog # 563313, BD Biosciences. PerCP-Cy5.5 Mouse Anti-Human IgM, clone MHM-88, 100 tests, Catalog # 314512 Biolegend. BV421-Mouse Anti-Human IgG, clone G18-145, 50 tests, Catalog # 562581 BD Biosciences. APC-Cy7-Mouse Anti-Human HLA-DR, clone L243, 100 tests, Catalog # 307618 Biolegend. BV480-Rat Anti-Human CXCR5, clone RF8B2, 100 tests, Catalog # 566142, BD Biosciences. Cells stained with the Spike Trimer were fixed with the transcription factor buffer (Thermofischer) and intra-cellularly stained for IRF4 (IRF4 Monoclonal Antibody (3E4), eFluor™ 660, eBioscience, Thermofischer) and Blimp-1 (BD Horizon™ PE-CF594 Rat Anti-Mouse Blimp-1, clone 6D3, 100tests, Catalog # 564269, BD Biosciences). ELISA kits were used according to manufacturer protocols and included antibodies as per Kit. design. From R&D Systems: Human CD14 DuoSet ELISA (DY383), Human CD163 DuoSet ELISA (DY1607), Human LBP DuoSet ELISA (DY870-05), Human Galectin-9 DuoSet ELISA (DY2045), Human GDF-15 Quantikine ELISA Kit (DGD150), Human CXCL4/PF4 Quantikine. ELISA (Kit DPF40), Human IFN-alpha (41100); from Ebioscience: Human MPO Instant ELISA Kit (BMS2038INST); from Thermo Scientific: Invitrogen novex IP 10 Human ELISA Kit (KAC2361); from Abcam Human C-Reactive Protein/CRP (Ab99995); from MyBioSource: Human zonulin ELISA Kit (MBS706368); from Meso Scale diagnostics: human Calprotectin (F21YB-3).

Validation

The antibodies were all confirmed in stains with blood bank sample PBMC and Veri-Cells Leukocytes control cells (from BioLegend). Ab working concentrations were titrated/confirmed as per manufacturor protocol as listed above.

Human research participants

Policy information about studies involving human research participants

Population characteristics

The cases are presented in Table 1 where mean and median age, sex, vaccination type (dose 2), time since infection, time since 2nd vaccine, time between 1st and 2nd dose and vaccine type (dose 1).

Recruitment

The cases were recruited as part of an outbreak investigation. Controls were included as part of established population cohorts and from healthy donors - volunteer health care workers. All subjects signed informed consent.

Ethics oversight

The study was approved by the Regulatory Ethics Commitee for South Eastern Norway (Approval numbers: REK 2021.233704; REK 2020.135924; REK 2021.229359).

Note that full information on the approval of the study protocol must also be provided in the manuscript.

Flow Cytometry

Plots

Confirm that:

☒ The axis labels state the marker and fluorochrome used (e.g. CD4-FITC).

☒ The axis scales are clearly visible. Include numbers along axes only for bottom left plot of group (a 'group' is an analysis of identical markers).

☒ All plots are contour plots with outliers or pseudocolor plots.

☒ A numerical value for number of cells or percentage (with statistics) is provided.

Methodology

Sample preparation

Standardised centrifugation of CPT tubes (PBMC, plasma) and serum

Instrument

BD FACSymphony (BD Biosciences) or Attune NxT (ThermoFisher).

Software

FlowJo v10 software (BD Life Sciences)

Cell population abundance

There was no cell sorting. 1M cells were stained for high dimensionality plots and HLA-dextramer staining to allow visualization of cells of low-abundance (0.1-0.01%).

Gating strategy

The analyses were both based on gating and unbiased with UMAP/phenograph functions as detailed in Figures. The step-by-step gating of standardised subsets is shown in the Supplementary data (Fig 8). TFH cells were identified in total CD4 T cells after exclusion of doublets, dead, non-T cells, gdTCR+ T cells and MAIT cells (CD161+TCR Va7.2+). Identification of Spike and Spike RBD SARS-CoV-2 binding B cells were identified in total B cells after exclusion of doublets, dead, and Lineage+ (Monocytes, NK cells and T cells). The phenotype of B cells was also studied by high parameters flow cytometry to detail naïve (IgD+) and Switched (IgG+) memory B cells. Examples of peptide:multimer stains are shown in Supplementary Figure 8.

☒ Tick this box to confirm that a figure exemplifying the gating strategy is provided in the Supplementary Information.
